# Efficient and stable carbon capture from industrial flue gas under humid conditions using amine-functionalized Zr-MOF-808 through statistical process design

**DOI:** 10.1039/d6ra07412j

**Published:** 2026-10-09

**Authors:** Neha Mohiuddin, Akshit Kumar, Rajesh Yadav, Satyapaul A. Singh, I. Sreedhar

**Affiliations:** a Department of Chemical Engineering, BITS Pilani Hyderabad Campus Hyderabad-500078 India; b R&D Centre, GAIL (India) Limited Noida U.P - 201301 India isreedhar@hyderabad.bits-pilani.ac.in +91 4066303998 +91 4066303512

## Abstract

Amine functionalization is a well-known approach to enhance CO_2_ adsorption capacity in metal–organic frameworks (MOFs), especially in humid environments. In this study, triethylenetetramine (TETA) was used to functionalize Zr-MOF-808 *via* wet impregnation, and its CO_2_ adsorption studies were performed using wet flue gas. To conduct the adsorption tests, a simulated industrial flue gas mixture was used, containing 12 vol% CO_2_, 10 vol% H_2_O, 8 vol% O_2_, with the remainder being N_2_. To systematically investigate the effects of various parameters on CO_2_ adsorption and identify optimal operating conditions, response surface methodology (RSM) combined with a central composite design (CCD) was employed. Under optimal conditions 55 °C temperature, 20 wt% TETA loading, 3 g of adsorbent, and 180 minutes of carbonation time, the model estimated an adsorption capacity of 3.6 mmol g^−1^, which is very close to the maximum experimental adsorption capacity of 3.4 mmol g^−1^. The RSM-CCD analysis indicated that both carbonation time and temperature significantly influence the results, while amine loading has a notable quadratic effect. The Avrami model provided the best fit for the kinetic behaviour, whereas the Sips isotherm most accurately described the isotherm behaviour. Moreover, Zr-MOF-808-TETA (20%) retained 88% of its original capacity after 10 consecutive cycles. Therefore, the results indicate that TETA-functionalized Zr-MOF-808 is a potential candidate for CO_2_ capture under humid simulated flue gas conditions.

## Abstract 2

Enhanced humid carbon capture through effective amine functionalization in MOF.

## Graphical abstract

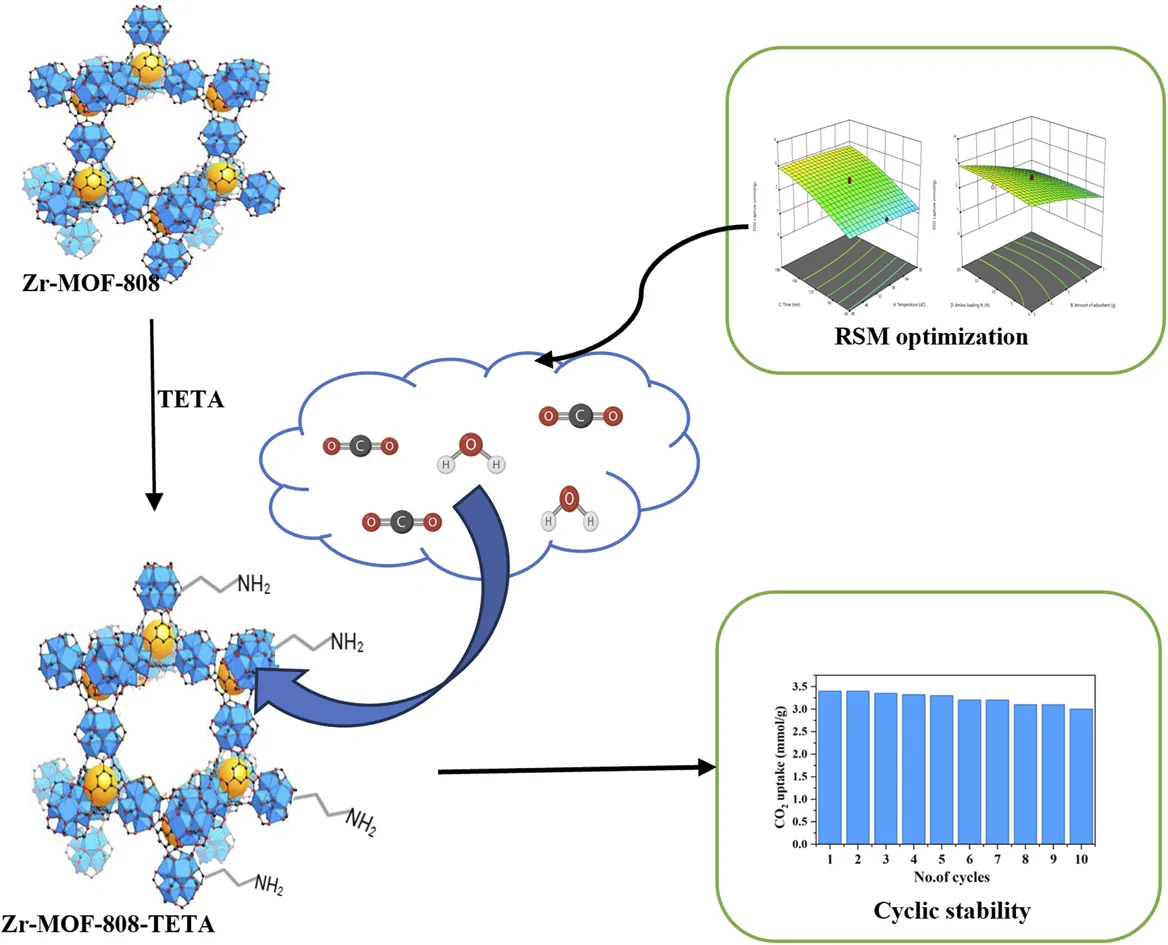

## 1 Introduction

Escalating levels of atmospheric CO_2_, driven primarily by industrial activities and fossil fuel combustion, have heightened global concerns about climate change and environmental damage. In response to these challenges, carbon capture and utilization (CCUS) has surfaced as an essential mitigation technology. As global CO_2_ emissions continue to rise, interest in carbon capture technologies has grown.^1^

The primary aim of Carbon Capture (CC) is to remove CO_2_ from emissions using methods such as pre-combustion, post-combustion, oxy-fuel combustion, and direct air capture (DAC).^2^ Post-combustion has emerged as an effective technology for CO_2_ capture, employing methods such as membrane separation, absorption, chemical looping combustion, cryogenic distillation and adsorption.^3^ Adsorption is employed in this study for its effectiveness, durability, and environmental friendliness.^4,5^

Various adsorbents employed in carbon capture include zeolites,^6^ carbon-based materials (activated carbon, biochar, graphene)^7,8^ zeolitic imidazole framework,^9^ covalent organic framework, metal–organic frameworks (MOF)^10^ and organic polymers.^11^ MOFs are frequently utilised in CC because of their customizable features, large surface area, exposed metal sites and pore volume.^10,12^ Recent studies continue to showcase the versatility of MOFs, which are widely used in adsorption, gas separation, water remediation and various environmental applications owing to their structural stability and tunability.^13,14^ In particular, recent developments in water-stable and hydrophobically modified MOFs have highlighted their potential for CO_2_ capture under humid and wet flue-gas conditions.^15^ MOF-based materials have also been extensively investigated through different modification strategies to improve their adsorption performance and stability.^16^ There are different synthesis methods employed in MOFs preparation, which include hydro/solvothermal, microwave-assisted, ultrasonication, and electrochemical. Past studies have found a broad spectrum of MOFs and their modifications.^10,17,18^ Libo Li and group have conducted comparative studies between M/DOBDC (M = Mg, Ni, Zn, Co, Mn) and other adsorbents, among which Mg/DOBDC has shown a good capture capacity of 248 cm^3^ g^−1^.^19^ The Mg/DOBDC MOF@GO that Wang and group synthesized exhibited an 8.30 mmol g^−1^ CO_2_ uptake, nearly twice that of its parent MOF.^20^

The key challenges in low-concentration CO_2_ capture in the presence of humidity are the structural decomposition of the MOF, reduced cyclic stability, lower affinity towards CO_2_, and MOF regeneration after multiple cycles. To overcome these difficulties, the emphasis has shifted to amine-modified MOFs, which exhibit high adsorption capacity under moisture-laden conditions. Amines, being basic, participate in acid–base interactions with CO_2_, thereby enhancing their affinity towards CO_2_.^21^ Amine modification involves using various amine precursors such as TEPA, PEI, and TETA.^22–27^ M. Anbia has modified MIL-101 with pentaethylenehexamine (PEHA), which showed 50% more CO_2_ uptake than the parent MOF.^28^ D. Zhang and group modified ZIF-8 using deep eutectic solvents, resulting in 13.71 times greater CO_2_ capture than parent ZIF-8.^29^ Liu and group synthesized 5A@MSA-30, a mesoporous silica-supported amine, which showed a high CO_2_ uptake of 5.05 mmol g^−1^ at 25 °C from humid flue gas.^30^ Z. Yonjia and his team performed a comparative analysis of bimetallic UiO-66 and UiO-66-NH_2_, using (Zr and Fe) as the metal centres, with adsorption capacities of 2.2 mmol g^−1^ and 3.5 mmol g^−1^, respectively.^31^

The novelty of this work lies in the deployment of the TETA-modified Zr-MOF to achieve successful carbon capture from humid flue gas through hierarchical and statistical process design. It examines the simultaneous effects of TETA loading and key parameters on CO_2_ adsorption in a mixture of 12 vol% CO_2_, 10 vol% H_2_O, 8 vol% O_2_, and N_2_, which reflects real flue gas conditions. RSM-CCD evaluates the linear, interaction, and quadratic effects of the parameters, as well as their overall contribution to adsorption. The study also investigates adsorption kinetics, equilibrium isotherms, and cyclic stability to characterize adsorption behaviour and regeneration efficiency of TETA-modified Zr-MOF-808 under humid conditions.

## 2 Experimental section

### 2.1 Materials and software employed

Zirconium oxychloride octahydrate (ZrOCl_2_·8H_2_O), trimesic acid (H_3_BTC), dimethyl formamide (DMF), formic acid, methanol, and triethylenetetramine (TETA) were all obtained from SRL chemicals at 99% purity and were used without further purification. Origin 2026 student version is used for graphical data visualization, while Design-Expert 11 is employed for process optimization.

### 2.2 Synthesis procedure

Initially, 0.3 g of trimesic acid and 0.7 g of ZrOCl_2_·8H_2_O were dissolved in 33.7 ml each of DMF and formic acid, then sonicated in an ultrasonic bath for 5 minutes. The solution was transferred to a Teflon-lined stainless-steel autoclave and heated at 130 °C for 48 hours. The resulting product was filtered, washed, and dried at 110 °C for 12 hours. The synthesis process is shown in Fig. 1. To prepare amine-modified Zr-MOF-808, 4 g of pristine Zr-MOF-808 was dispersed in 25 ml of methanol, and for a 10 wt% TETA loading, 0.44 g of TETA was dissolved in 25 ml of methanol. Both solutions were stirred at room temperature for 12 hours. The resulting solid was washed once with methanol and twice with DI water, filtered, and dried at 80 °C in a vacuum oven for 6–8 hours. The same method was used for 20 wt% TETA loading, in which 1 g of TETA was dissolved in 50 ml of methanol and 4 g of Zr-MOF-808 in 25 ml of methanol. The mixture was stirred at room temperature for 12 hours, then washed with methanol once and DI water twice, filtered, and dried at 80 °C in a vacuum oven for 6–8 hours. The TETA loading was calculated by using eqn (1). TETA loadings of 10 and 20 wt% refer to the nominal mass fraction of TETA in the total TETA-Zr-MOF-808 mixture. The 10 and 20 wt% values represent the nominal TETA loadings, which may differ from the actual amount of TETA retained after impregnation and washing due to potential TETA loss during washing and drying.1
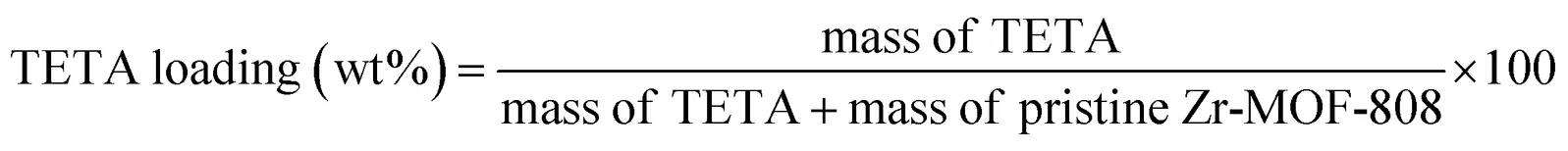


**Fig. 1:**
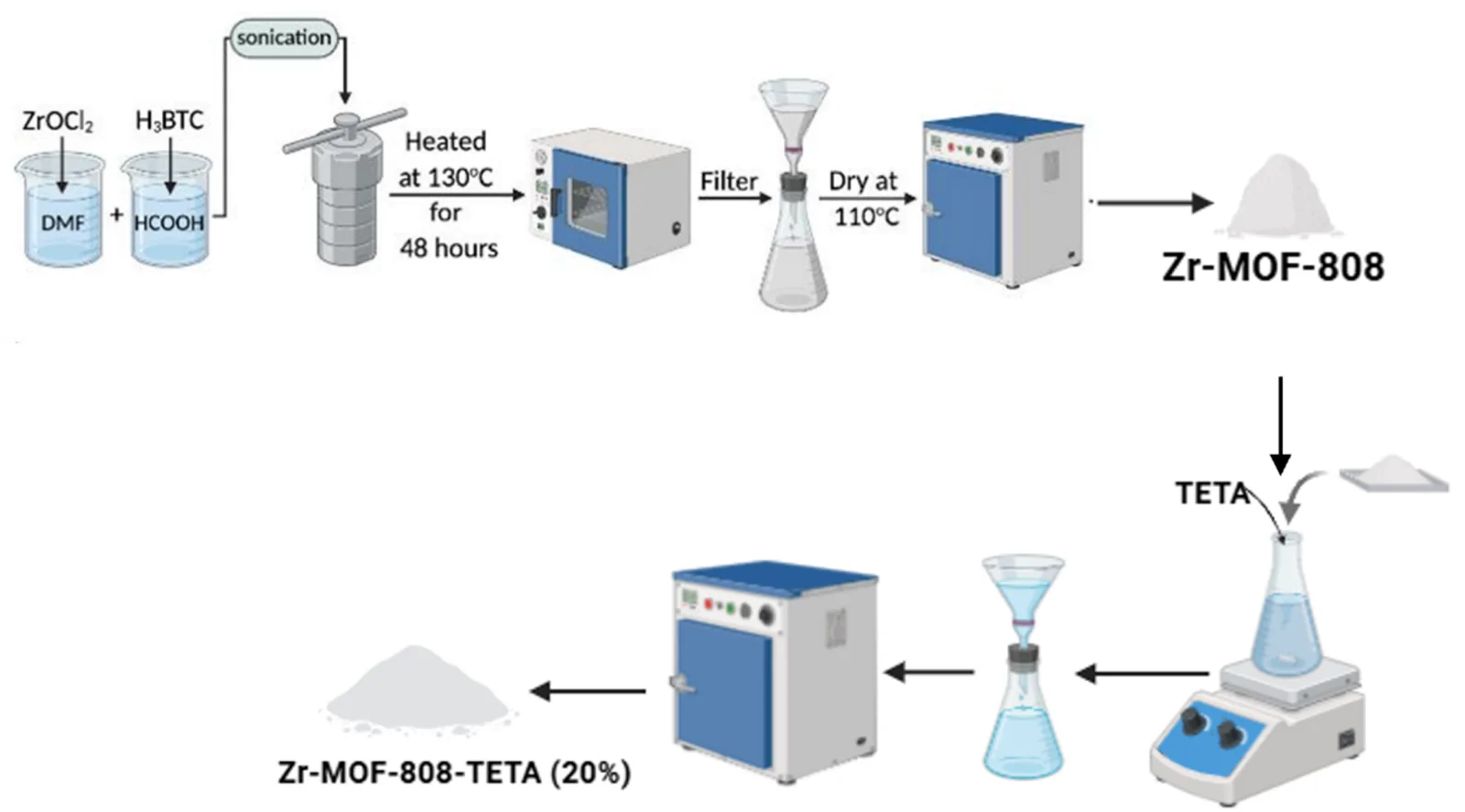
Synthesis procedure.

### 2.3 Physicochemical characterization studies

#### 2.3.1 Structural and morphology studies

Powder X-ray diffraction (PXRD) was conducted using the Rigaku ULTIMA-IV model with a Cu X-ray source (*λ* = 0.154 nm) to analyze phase formation and crystallinity over a 2*θ* range of 5–40°. Field-emission scanning electron microscopy (FE-SEM) was conducted using the FEI Apreo LoVac, which features integrated STEM and a DBS detector to examine the surface morphology of the adsorbents. Energy-dispersive X-ray spectroscopy (EDS) mapping using Aztec (Oxford Instruments) was used to examine the elemental composition of the synthesized adsorbents. Before analysis, samples were sputter-coated with gold. The measurements were conducted under high-vacuum conditions at an accelerating voltage of 15 kV and a working distance of about 10 mm. The surface area and pore volume were measured with N_2_ adsorption isotherms using a Microtrac BEL SORP mini II instrument, and the data were analysed with the BEL SORP mini software.

#### 2.3.2 Functional group studies

The various functional groups were analyzed using FTIR with a Jasco P-200 spectrometer, which operated between 400–4000 cm^−1^ wavenumbers, providing high-resolution and accurate wavenumber measurements.

#### 2.3.3 X-ray photoelectron spectroscopy (XPS)

X-ray photoelectron spectroscopy (XPS) by Thermo Fisher Scientific, USA, featuring an Al anode source for identifying various elements and their oxidation states.

#### 2.3.4 Thermal stability analysis

Thermogravimetric analysis (TGA) using a Setaram Themys one + that can reach up to 1600 °C at ramp rates of 0.01 to 100 °C min^−1^. Here, samples are heated in an alumina sample holder from 30 °C to 800 °C at 10 °C min^−1^ under a nitrogen flow rate of 100 ml min^−1^.

### 2.4 Experimental setup

A lab-scale fixed-bed reactor configuration was used to perform CO_2_ adsorption studies under dry and humid environments. The experimental apparatus has a reactor bed with a length of 0.52–0.58 m and an inner diameter of 0.02–0.025 m. The reactor, packed with the adsorbent, was housed in an electric furnace with temperature monitored using a thermocouple. Inlet gas stream velocities were regulated *via* mass flow controllers (MFC). The gas flows through the reactor bed, then through the condenser and moisture trap, before reaching the gas detector, which monitors the CO_2_ levels in the effluent. Gas mixture consists of 12 vol% CO_2_, 10 vol% H_2_O, 8 vol% O_2_ and balance N_2_. The RH% at optimum conditions, *i.e.*, 55 °C, is 64.3%, and the water partial pressure is 10.13 kPa. The flow rate of the gas mixture is 100 ml min^−1^. The reactor configuration is shown in Fig. 2.

**Fig. 2:**
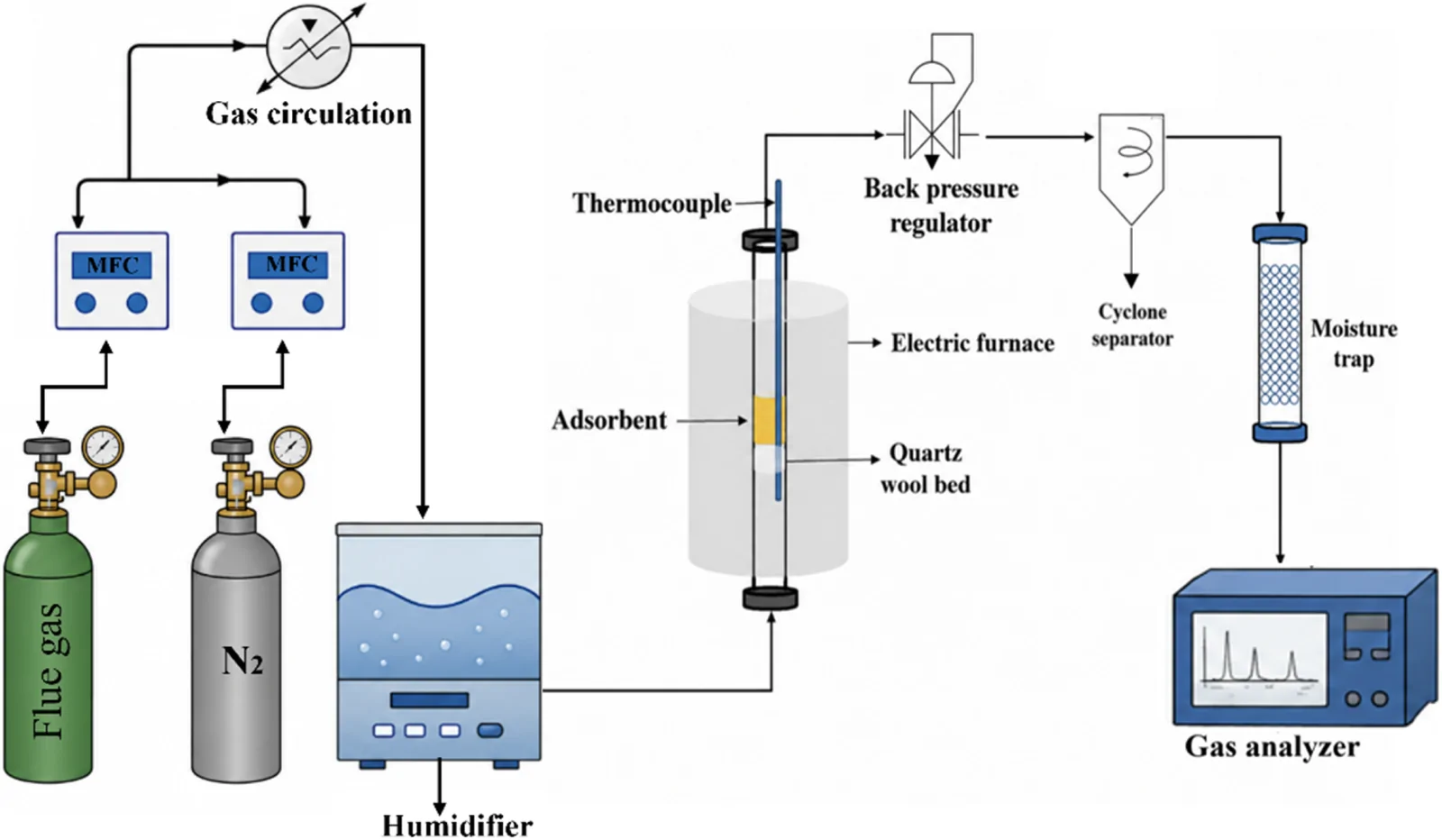
Fixed-bed reactor for carbon capture studies.

## 3 Characterization studies

### 3.1 XRD analysis

The PXRD analysis was used to assess the phase formation and crystallinity of pristine Zr-MOF-808 and the amine-functionalized MOF. The respective diffractograms are shown in Fig. 3. The significant reflections observed at 2*θ* values of 8.27°, 8.71°, 10.03°, and 10.91° correspond to the crystalline planes (311), (222), (400), and (331), respectively. The following pattern is consistent with the reported data for Zr-MOF-808.^32,33^ This suggests that the desired framework has been successfully synthesized. After amine modification, the peak at 8.27° decreased in intensity. However, the overall peak positions remained unchanged, indicating retention of the parent framework. The crystallite sizes of the following samples were calculated using the Debye–Scherrer equation, based on the most intense peak.^34^ The obtained crystallite sizes are 11.37 nm for pristine Zr-MOF-808, 11.02 nm for Zr-MOF-808-TETA (10%), and 10.16 nm for Zr-MOF-808-TETA (20%).

**Fig. 3:**
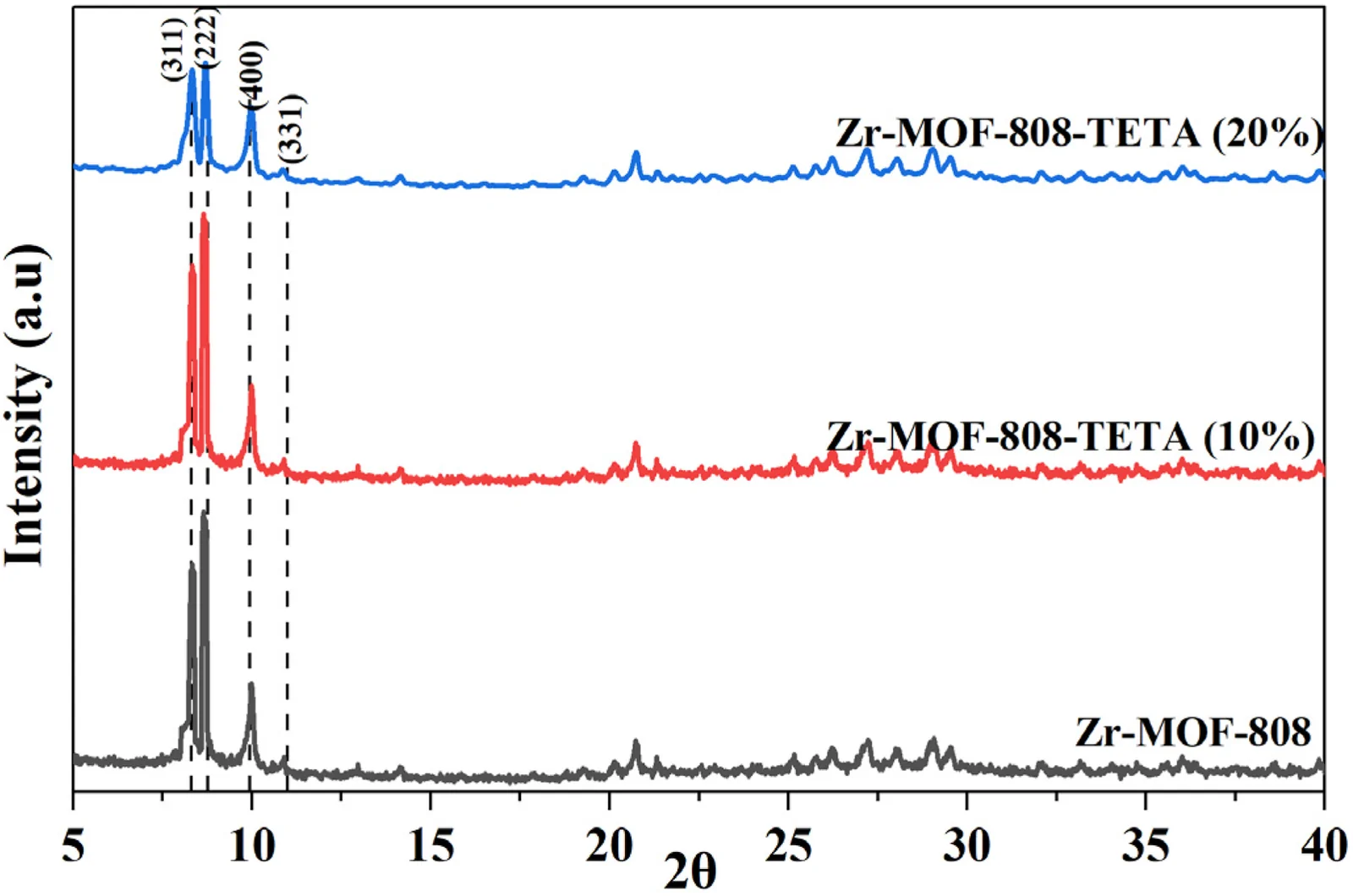
XRD diffractogram of Zr-MOF-808 and amine-modified Zr-MOF-808.

### 3.2 FTIR spectra

FTIR analysis was performed to identify the functional groups present in the adsorbent, with the spectra shown in Fig. 4. The spectra show characteristic vibrational bands confirming the framework structure and successful incorporation of TETA into amine-modified Zr-MOF-808. The pristine Zr-MOF-808 displays notable peaks at 500 cm^−1^ and 650 cm^−1^, corresponding to Zr–O–Zr and Zr–OH vibrations. Additionally, the peak between 3000 and 3700 cm^−1^ indicates Zr–OH and –OH vibrations.^35,36^ Few significant changes were observed after incorporating TETA, suggesting successful amine functionalization of the MOF. The Zr-MOF-808-TETA samples showed peaks at 1450–1470 cm^−1^, linked to CH_2_ bending; bands in the 1500–1600 cm^−1^ range, related to N–H bending of the amines; and broader peaks from 3200–3600 cm^−1^, representing secondary N–H stretching vibrations of amines, along with OH groups from water molecules adsorbed in the MOF framework.^37^ The intensity of this broad band increased with TETA loading, particularly for the 20% TETA sample, suggesting greater incorporation of amine species.

**Fig. 4:**
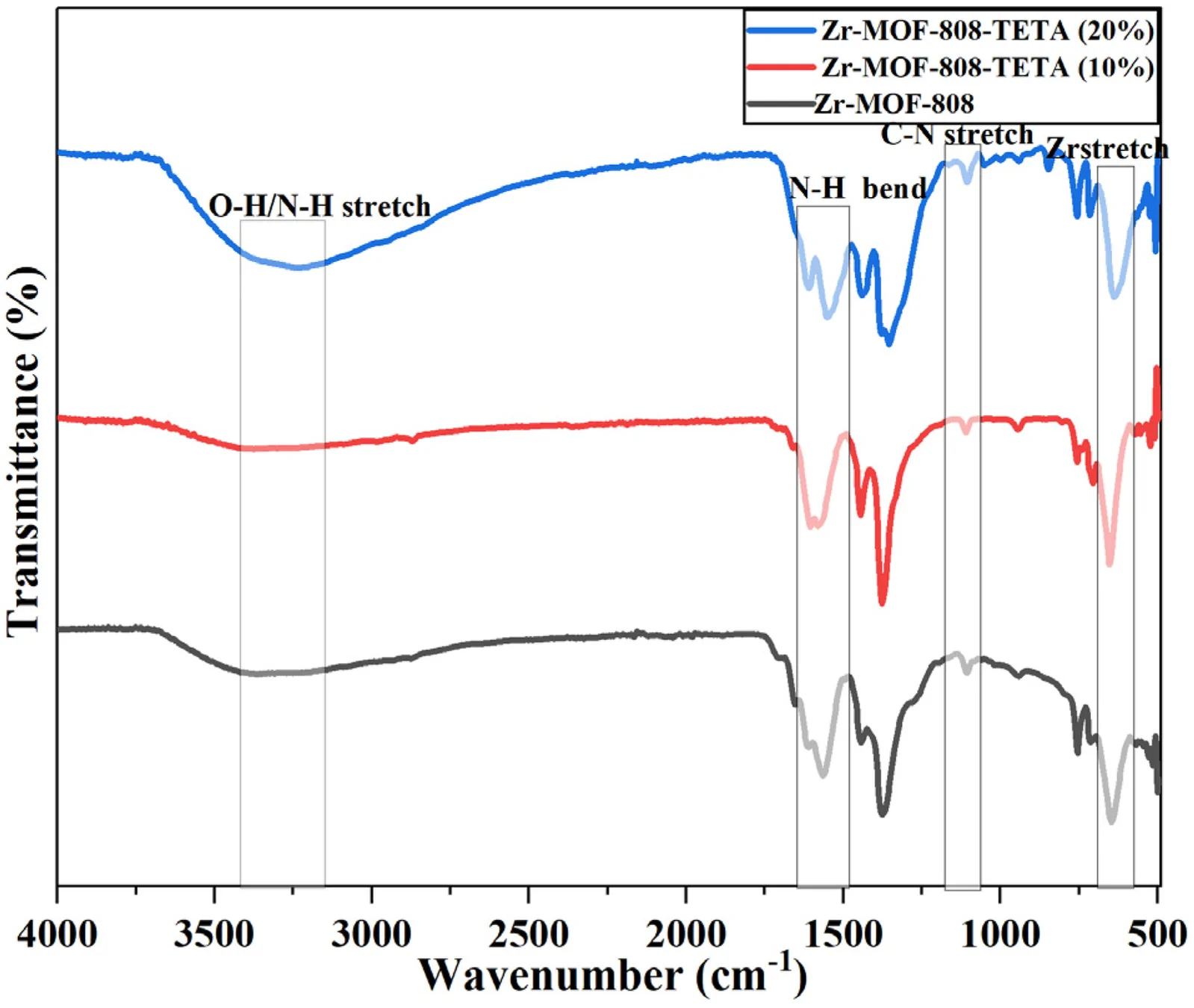
FTIR spectra of pristine and amine-modified Zr-MOF-808.

### 3.3 Morphology analysis

FE-SEM analysis examined the surface morphology of pristine Zr-MOF-808 and TETA-functionalized Zr-MOF-808, shown in Fig. 5. All images have a scale bar of 500 nm. The pristine Zr-MOF-808 features densely packed, nearly spherical particles with a rough surface texture and evident particle agglomeration. After TETA functionalization, the overall particle shape remained similar, but particles showed increased agglomeration and smoother, more compact surfaces, suggesting TETA deposition on the external surface of the MOF. At 10 wt% TETA loading, the sample resembled pristine Zr-MOF-808, indicating that moderate amine loading had little impact on particle shape. In contrast, at 20 wt% TETA loading, particles exhibited dense packing, increased surface coverage, and agglomeration, indicating greater TETA deposition while maintaining the general particle morphology. These morphological findings are supported by the XRD results, which indicated a decrease in intensity without new peak formation, indicating the MOF framework was preserved with only a slight reduction in crystallinity after TETA impregnation. The average particle size of pristine Zr-MOF-808 was determined to be 453.9 ± 71.64 nm. The Zr-MOF-808-TETA (10%) and (20%) samples had mean sizes of 498.15 ± 74.58 nm and 516.62 ± 79.53 nm, respectively. The increase in the observed mean particle size after TETA modification suggests that TETA may influence particle agglomeration and the apparent particle size. All particle size measurements are based on SEM images taken at 500 nm and are calculated using ImageJ software. The resulting particle-size distribution data are shown in Fig. S5.

**Fig. 5:**
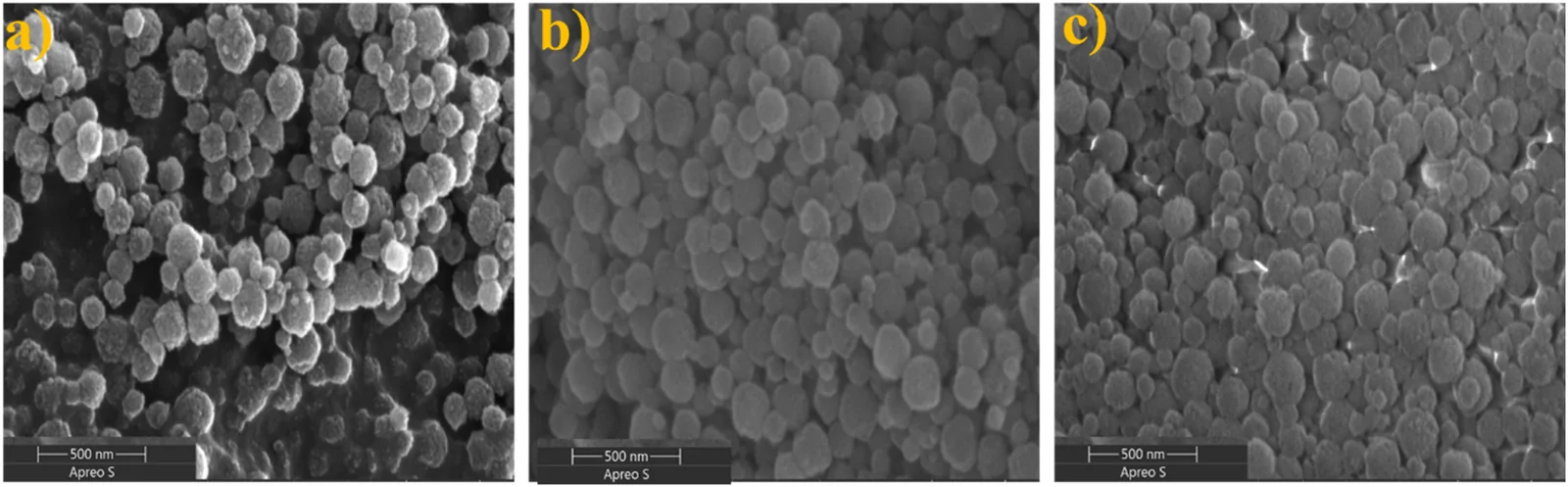
FE-SEM micrographs of (a) pristine Zr-MOF-808, (b) Zr-MOF-808-TETA (10%), and (c) Zr-MOF-808-TETA (20%).

Both EDS spectra and the corresponding elemental weight percentages for all materials are presented in the accompanying Fig. S6 and Table S1. Elemental analysis confirms the presence of Zr, N, O, and C. Nitrogen content increased from 0.03 wt% in pristine Zr-MOF-808 to 2.71 and 5.90 wt% in the 10 and 20 wt% TETA-modified samples, respectively, consistent with the incorporation of nitrogen-containing TETA species through amine functionalization. Zr, C, and O remained detectable in all samples, indicating that the characteristic elemental composition of the Zr-MOF-808 framework was retained after TETA modification. The spatial distribution and elemental composition of the pristine and TETA-modified Zr-MOF-808 were investigated using SEM-EDS elemental mapping, as shown in Fig. S7. For all samples, Carbon (C, red), Oxygen (O, cyan), Nitrogen (N, green), and Zirconium (Zr, purple) were analyzed. The elemental maps exhibited a relatively homogeneous spatial distribution, with no prominent isolated regions of high elemental intensity. This observation suggests a relatively uniform distribution of the TETA-associated elemental signals without the formation of large TETA-rich agglomerates. Pristine Zr-MOF-808 showed a weak N signal, while amine modification with TETA produced a distinct, clear N signal. The N-map density also increased stepwise from 10 wt% to 20 wt% TETA loading, indicating greater incorporation of amine functionalities at higher nominal TETA loading.

### 3.4 Textural property analysis

N_2_ adsorption–desorption isotherms were measured at 77 K to analyse the surface area and porosity of the adsorbents. The isotherms are presented in Fig. 6a, while the related textural properties are detailed in Table 1. Pristine Zr-MOF-808 has a surface area of 779.05 m^2^ g^−1^ and a pore volume of 0.9004 cm^3^ g^−1^. When functionalized with TETA, both the BET surface area and pore volume of Zr-MOF-808-TETA (10%) gradually decreased to 580.97 m^2^ g^−1^ and 0.7164 cm^3^ g^−1^, respectively. In comparison, Zr-MOF-808-TETA (20%) showed a lower surface area of 145.95 m^2^ g^−1^ and a pore volume of 0.3254 cm^3^ g^−1^. This steady decline in surface area and pore volume indicates that TETA molecules partially fill the internal pores, blocking access to pore channels. All adsorbents have an average pore diameter greater than 4 nm, indicating a mesoporous structure. However, Fig. 6b shows that the main pore size remained near 2.1 to 2.5 nm after TETA functionalization. Although the calculated average pore diameter increased from 4.62 to 8.92 nm with higher TETA loading, this increase likely results from smaller pores being more occupied by TETA molecules. The reduction in both surface area and pore volume verifies pore blockage following amine impregnation.

**Fig. 6:**
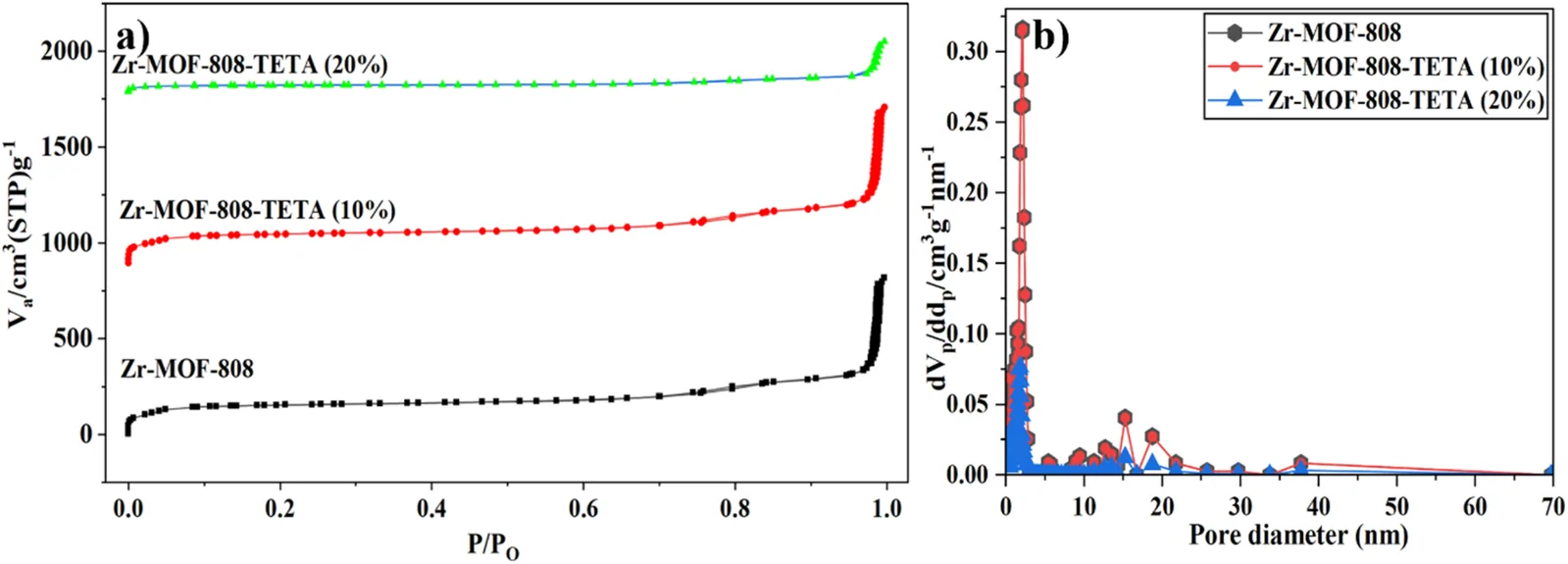
(a) N_2_ adsorption–desorption isotherms of Zr-MOF-808 and amine-modified Zr-MOF-808, and (b) NLDFT pore-size distribution curves.

**Table 1:** BET analysis of pristine and amine-modified Zr-MOF-808

S. no.	Name of the adsorbent	Surface area (m^2^ g^−1^)	Total pore volume (cm^3^ g^−1^)	Average pore diameter (nm)	NLDFT pore size (nm)
1	Zr-MOF-808	779.05	0.9004	4.623	2.1
2	Zr-MOF-808-TETA (10%)	580.97	0.7164	6.1994	2.3
3	Zr-MOF-808-TETA (20%)	145.95	0.3254	8.9182	2.5

A similar decrease in surface area is seen during amine modification.^35,37,38^ The surface area of M-808 decreased from 1706 to 814 and 20 m^2^ g^−1^ upon TEPA and DETA loading on the MOF-808 surface.^39^ Similarly, the surface area of HKUST-1 decreased from 708 to 327 m^2^ g^−1^ upon TEPA modification. Similarly, the ZIF-8 surface area decreased from 1742 to 1074 m^2^ g^−1^ upon TEPA impregnation.^40^

### 3.5 Thermogravimetric analysis (TGA)

Thermogravimetric analysis (TGA) and differential thermal analysis (DTA) were conducted on both pristine and TETA-functionalized Zr-MOF-808 to assess their thermal stability and decomposition patterns. The samples were heated from 30 °C to 800 °C at a rate of 10 °C min^−1^ in presence of N_2_ gas, and the resulting thermograms are shown in Fig. 7. Pristine Zr-MOF-808 showed an initial weight loss up to approximately 100 °C, retaining 83.8% of its original mass, due to the removal of physically adsorbed moisture and residual solvent molecules from the porous structure.^41^ A gradual mass decrease between 100 and 350 °C indicates partial decomposition of the coordinated organic linkers, yielding a residual mass of 67.15%. The most significant weight loss occurs above 350 °C, when the MOF structure degrades to form ZrO_2_, leaving a residual mass of 43.8% at 620 °C.^42^ The thermal decomposition behaviour is in good agreement with previously reported studies on Zr-MOF-808,^43,44^ while the relatively high residual mass reflects the excellent thermal stability of this zirconium-containing MOF structure.

**Fig. 7:**
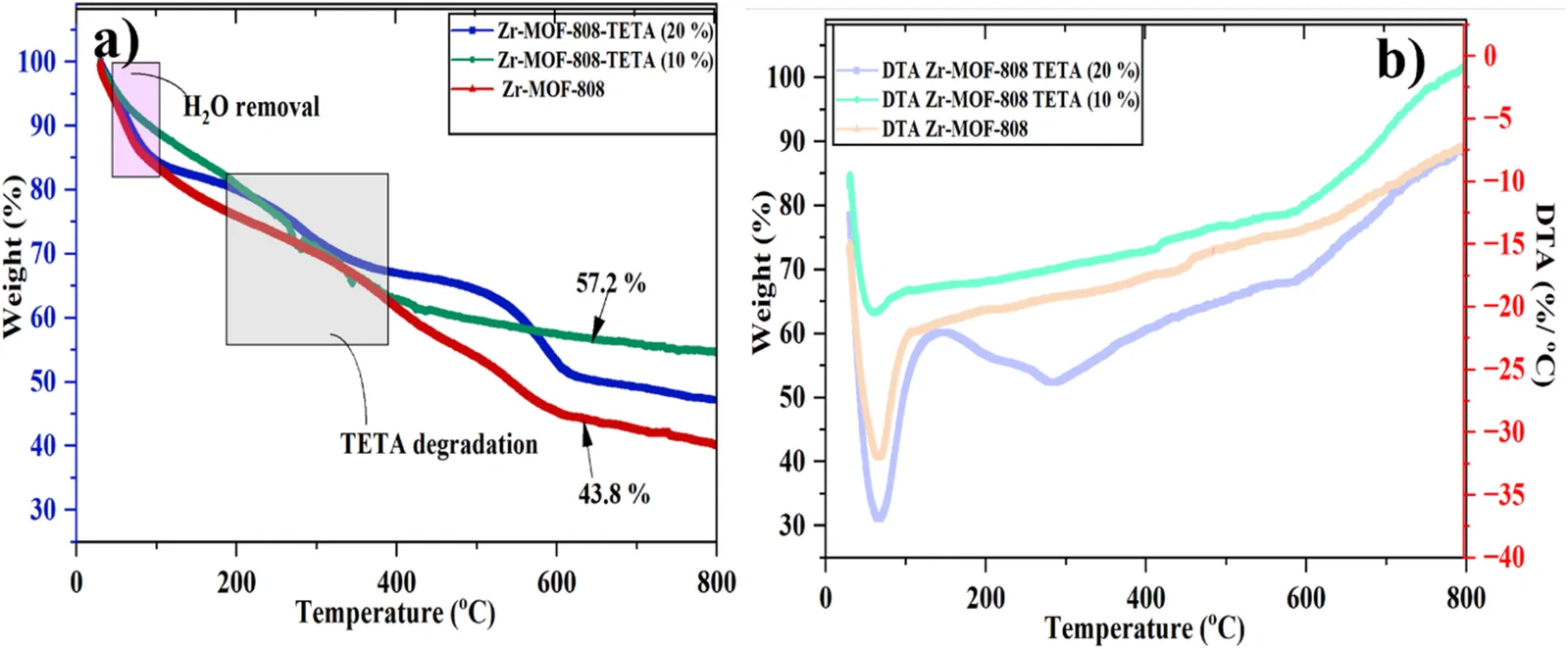
(a) and (b). TGA and DTA of pristine and amine-modified Zr-MOF-808.

In the amine-modified samples, additional mass loss was noted because of the decomposition of impregnated TETA (triethylenetetramine) molecules. Zr-MOF-808-TETA (10%) maintained 88.6% of its original weight up to 110 °C, which indicates the evaporation of physically adsorbed moisture and volatile substances. As the temperature increased further, the remaining mass decreased to 61.5% at 436 °C and to 57.2% at 630 °C. Zr-MOF-808-TETA (20%) retained 84.1% of its original weight up to 100 °C, followed by a gradual decrease to 68.86% at 355 °C, and 50.85% at 630 °C. The greater weight loss in amine-functionalized samples confirms TETA's incorporation into the porous framework, and the decreasing residual mass with higher amine loading suggests more thermally decomposable organic amine groups are present within the adsorbent.

The DTA data also supported the TGA results by showing endothermic and decomposition attributed to solvent evaporation, amine degradation, and framework degradation. Despite these changes, both modified samples remained thermally stable up to about 300–350 °C, highlighting their potential for post-combustion CO_2_ capture at moderate temperatures. TGA determined TETA incorporation in Zr-MOF-808 by comparing modified and unmodified MOF mass loss. TETA loadings were 0.1986 mmol g^−1^ at 10 wt% and 0.4506 mmol g^−1^ at 20 wt%. Based on BET surface areas, surface nitrogen densities were 0.00171 and 0.01544 mmol of N m^−2^, respectively.

### 3.6 X-ray photoelectron spectroscopy (XPS)

The X-ray photoelectron spectroscopy (XPS) survey spectra of both pristine and TETA functionalized Zr-MOF-808 samples are shown in SI data Fig. S1–S4. These spectra verify the presence of Zr, O, N, and C elements in all adsorbents. Additionally, the appearance of the N 1s peak in Zr-MOF-808-TETA (at 10% and 20%) indicates successful integration of TETA into the framework. The Zr 3d spectra exhibit two characteristic peaks, representing Zr 3d_5/2_ and Zr 3d_3/2_,^41^ indicating that the zirconium coordination environment is preserved after amine functionalization. However, a slight shift in binding energy from 182.38 to 182.08 eV for Zr 3d_5/2_ and 185.08 to 184.52 eV for Zr 3d_3/2_ of the TETA-modified samples suggests electronic interaction between amine groups and Zr metal centres. Furthermore, the N 1s spectra confirm the presence of nitrogen-containing amine functionalities introduced through TETA introduction. The N peak appears at a slightly lower value, between 399.5 and 399.8 eV, indicating the presence of free amine groups in the framework.^45^ The O 1s spectra exhibited 3 peaks attributed to Zr–O, Zr–OH, and C=O.^42^Fig. 3 displays the FTIR spectra, clearly indicating the presence of these functional groups.

## 4 Process optimization using RSM

RSM is employed to develop a statistically sound experimental design to investigate how key operating parameters influence CO_2_ adsorption. A central composite design (CCD), one of the most commonly applied RSM approaches, was adopted to examine the single, combined and quadratic impacts of the selected variables on the response.^46^ The CCD contains central, factorial and axial points. Four key process parameters, namely temperature, carbonation time, % amine loading, and adsorbent mass, were chosen as independent variables and examined at three levels (−1, 0, +1). The ranges and levels for these variables are detailed in Table 2. The coded (−1, 0, +1) levels represent the factorial and centre levels, respectively, while additional axial/extended experimental points were included beyond the factorial region for selected factors. The overall ranges were 3–10 g adsorbent loading, 60–240 minutes carbonation time, 30–85 °C temperature, and 0–30 wt% TETA loading. This statistical method aids in understanding the experiment and identifies optimal conditions to improve CO_2_ adsorption. A total of 30 experimental runs were conducted to assess the impact of each parameter on CO_2_ uptake. The outcomes of these runs are included in the SI data. Table S2. All experiments were conducted under humid conditions.

**Table 2:** Different parameters for RSM studies

Parameters	Units	−1	0	+1	Axial points
Amount of adsorbent loading	g	3	5	7	10
Carbonation time	min	60	120	180	240
Temperature	°C	40	55	70	85
wt% of amine loading	wt%	0	10	20	30

### 4.1 Influence of operating parameters on CO_2_ adsorption

The study analysed parameters such as temperature, amine loading, carbonation time, and adsorbent amount. Notably, higher temperatures reduced CO_2_ adsorption capacity, indicating an inverse relationship. This trend is consistent with the exothermic nature of the adsorption process, in which higher temperatures increase the kinetic energy of CO_2_ molecules and diminish their interactions with the adsorbent surface.^47,48^ Previous studies on MOF-based adsorbents showed a similar trend: higher temperatures led to reduced CO_2_ uptake due to reduced physisorption and the destabilisation of the adsorbent structure.^49^ In contrast, increased amine loading significantly improved adsorption performance by increasing adsorption sites, thereby enhancing adsorbent efficiency towards CO_2_. This improvement aligns with the known mechanism of chemisorption involving the amine groups of CO_2_ molecules. However, excessive amine loading may eventually lead to pore blockage and reduced accessibility to active sites, as reported in several earlier investigations on amine-functionalized porous adsorbents.^50^ The carbonation time positively influenced CO_2_ adsorption capacity, though the increase was modest. Longer durations increase contact between gas molecules and the adsorbent surface, thereby enhancing diffusion and adsorption within the porous structure. In this study, the amount of adsorbent loaded also influenced the CO_2_ adsorption capacity. While a small decrease in CO_2_ adsorption was observed as the adsorbent mass increased, the overall effect was statistically significant. This could be due to competing phenomena, such as increased availability of adsorption sites on the one hand and reduced gas–solid interaction efficiency due to particle stacking and diffusion limitations on the other. Overall, ANOVA results indicates that the carbonation time and the amount of adsorbent are the primary factors influencing CO_2_ adsorption performance, whereas the linear effects of temperature and amine loading were not statistically significant (*p* > 0.05); however, the significant quadratic effect of amine loading (*D*^2^) indicates a nonlinear response and suggests the presence of an optimum amine loading within the investigated range under the conditions studied according to the *F*-value obtained from ANOVA results presented in Table S3. In this study, the influence of temperature (*A*), adsorbent amount (*B*), carbonation time (*C*), and % amine loading (*D*) on CO_2_ adsorption was evaluated using response surface and contour plots generated from the quadratic RSM model. The *AC* plot depicts that CO_2_ uptake decreases with increasing temperature but increases with longer carbonation time. The *BD* plot shows the amount of adsorbent and the wt% amine loading, with moderate amine incorporation enhancing CO_2_ uptake, whereas excessive loading led to pore blockage and diffusion limitations. Fig. 8 displays the following contour plots.

**Fig. 8:**
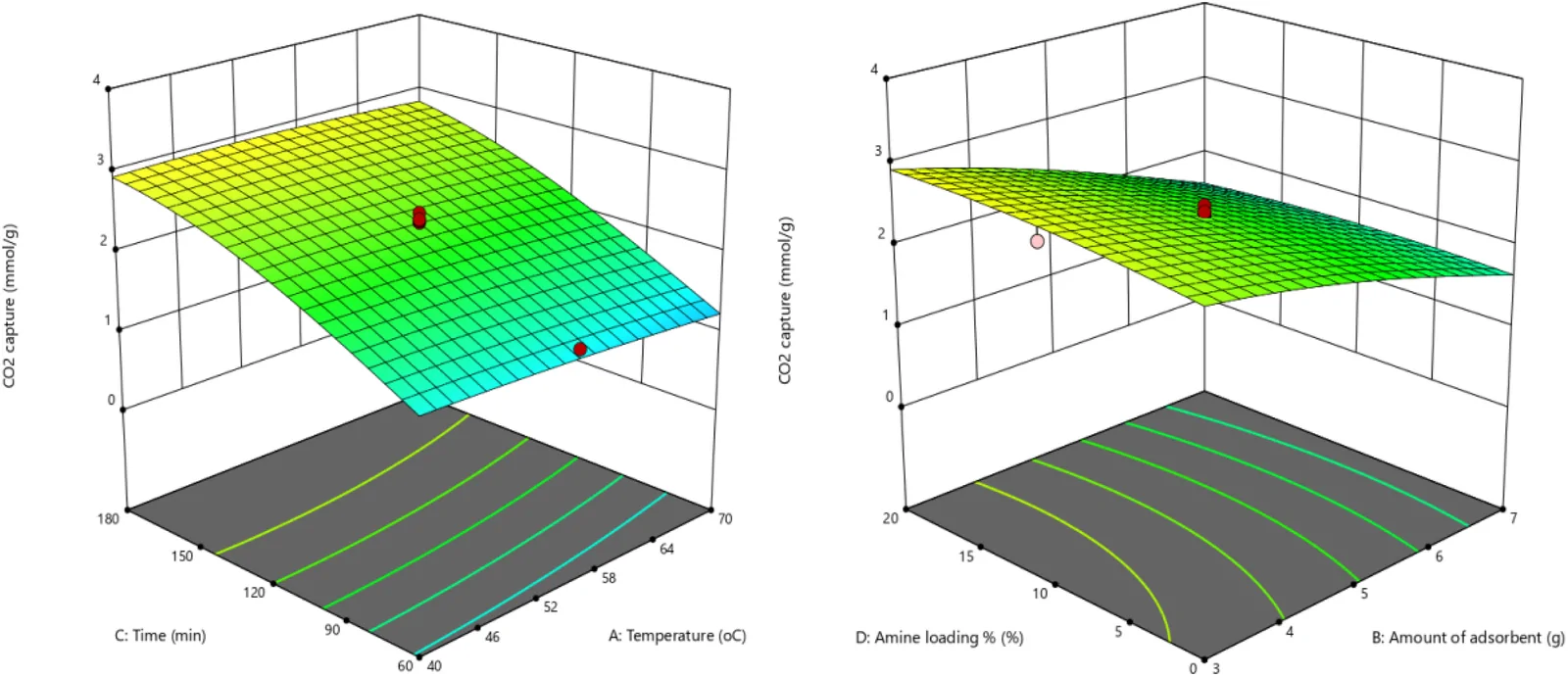
Visual depiction of the interaction between various parameters influencing CO_2_ adsorption capacity.

### 4.2 RSM validation through ANOVA and fit statistics

The quadratic equation derived from the RSM-CCD model describing the relationship between CO_2_ adsorption and the process parameters studied is presented below.CO_2_ adsorption capacity (mmol g^−1^) = 1.48 − (0.0232 × *A*) − (0.4527 × *B*) + (0.2590 × *C*) + (0.0023 × *D*) − (0.0084 × *AB*) + (0.0391 × *AC*) + (0.0143 × *AD*) + (0.0153 × *BC*) − (0.0462 × *BD*) + (0.0308 × *CD*) − (0.0267 × *A*^2^) − (0.1327 × *B*^2^) − (0.2055 × *C*^2^) − (0.0245 × *D*^2^).Here, *A* is the temperature (°C), *B* is the amount of adsorbent (g), *C* is the carbonation time (min), and *D* is the % amine loading (wt%). The regression equation is expressed in terms of coded, dimensionless variables corresponding to the experimental factor levels used in the CCD.

The model was highly significant, with an overall *F*-value of 94.32 and a *p*-value less than 0.0001, demonstrating that the quadratic model adequately described the relationship between the investigated process parameters and the adsorption response.

Table 3 outlines the optimal conditions identified by RSM and the corresponding experimental and predicted CO_2_ uptake values. To improve precision, the optimal condition is tested three times, yielding an experimental value of 3.4 mmol g^−1^ (standard deviation: ±0.20), which is closer to the predicted value of 3.6 mmol g^−1^.

**Table 3:** Optimum conditions obtained through the RSM model

S. no.	Temperature (°C)	Amine loading (wt%)	Amount of adsorbent loading (g)	Time of carbonation (min)	Experimental CO_2_ uptake (mmol g^−1^)	Predicted CO_2_ uptake (mmol g^−1^)
1	55	20	3.00	180	3.4 ± 0.20	3.6

While the ANOVA results demonstrate that among the investigated variables, carbonation time showed the strongest impact on CO_2_ adsorption, with the highest *F*-value of 359.63 and a *p*-value below 0.0001, followed by the amount of adsorbent, with an *F*-value of 125.39. This behaviour indicates that prolonged gas–solid interactions and an increased number of active adsorption sites significantly enhance adsorption performance. The linear effect of temperature was not statistically significant within the investigated range (*p* = 0.2850), while the linear effect of amine loading was also not significant (*p* = 0.8838). However, the quadratic term of amine loading (*D*^2^) was significant (*p* = 0.0446), suggesting a nonlinear response and the presence of an optimum amine incorporation level. The *BD* interaction between the amount of adsorbent and amine loading was significant, indicating that these parameters jointly affect the adsorption response. Furthermore, the pronounced nonlinear effects of the quadratic terms (*B*^2^, *C*^2^, and *D*^2^) confirm curvature across the response surface. These findings indicate that the quadratic model adequately describes the experimental response and is suitable for process optimization. Its effectiveness was confirmed by a lack-of-fit test, which was not statistically significant (*p* = 0.1160), showing good agreement between the experimental data and the predicted equation. The robustness of the model is also supported by the goodness-of-fit statistics in Table S3, with an *R*^2^ of 0.9888. The adjusted *R*^2^ (0.9783) and predicted *R*^2^ (0.8933) indicated acceptable predictive capability. The low standard deviation (0.0444) and low CV% (3.15) further support the precision, reproducibility, and robustness of the RSM model. To further assess the adequacy of the developed quadratic model and verify the statistical assumptions of ordinary least squares regression, intensive residual diagnostic tests were conducted using externally studentized residuals, as shown in Fig. S8. As shown in Fig. S8a, the error points align closely with the straight diagonal line, indicating that the normality assumption is satisfied. The plot of studentized residuals against predicted values in Fig. S8b shows points randomly scattered around the zero line, indicating constant variance. Potential time trends and bias from individual factors were assessed by plotting residuals against run order as shown in Fig. S8c and individual candidate in Fig. S8d.

ANOVA, fit statistics, and coefficients in terms of coded factors are provided in the SI Tables S3–S5.

### 4.3 Adsorption kinetic studies

Kinetic modelling describes the adsorption behaviour and provides information on the adsorption rate and possible mass-transfer characteristics of the process. In this study, kinetic experiments were performed to evaluate the suitability of different kinetic models for describing CO_2_ uptake by Zr-MOF-808-TETA under optimal conditions identified by the RSM-CCD model. Generally, the pseudo-first-order (PFO) model describes adsorption based on the availability of adsorption sites, whereas the pseudo-second-order (PSO) model assumes that the adsorption rate depends on the second-order rate expression. The Avrami model provides an empirical description of uptake profiles that may involve different kinetic regimes, whereas the Elovich model describes various adsorption types on heterogeneous sites. The Weber–Morris model is designed to identify the specific stages of mass-transfer resistance during the adsorption process. The Bangham model evaluates whether adsorption is controlled by internal pore volume and structural features, assuming a uniform pore system. Boyd's diffusion model assesses the contributions of external-film and intraparticle diffusion to the overall adsorption rate between film and particle diffusion, with the latter as the rate-limiting step, relying on assumptions of spherical geometry and a homogeneous solid state. The respective equations are given in eqn (2)–(8).^51,52^ Kinetic fits alone cannot establish adsorption mechanisms: PFO and PSO do not prove physisorption or chemisorption. Mechanistic interpretation also requires isotherms, physicochemical characterization, and thermodynamic evidence, such as isosteric adsorption heat. Kinetic models mainly describe adsorption behaviour and rates.

2 *Q*_*t*_ = *Q*_e_(1 − e^−*k*_1_*t*^)

3
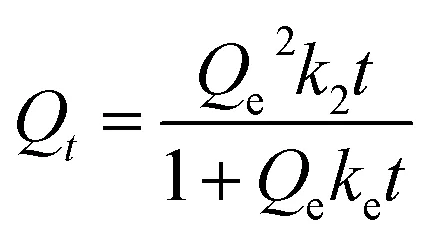

4 *Q*_*t*_ = *Q*_e_(1 − e^(−*k*_a_*t*^*n*^)^)

5
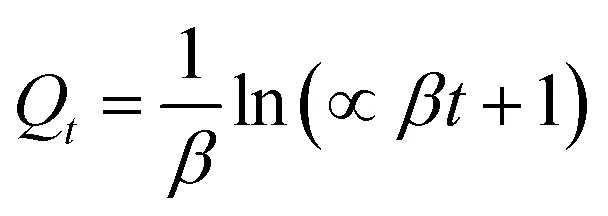
6
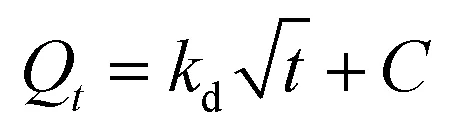

7 *Q*_*t*_ = *Q*_e_(1 − exp(−*k*_B_ × *t*^2^))

8
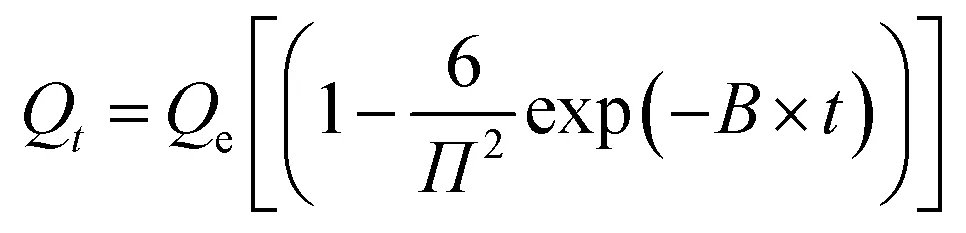
where *Q*_*t*_ and *Q*_e_ denote the CO_2_ uptake (mmol g^−1^) at time *t* and at equilibrium, respectively. The constants *k*_1_, *k*_2_, and *n* represent PFO, PSO, and the Avrami kinetic exponent. In the Elovich model, *α* denotes the initial adsorption rate, while *β* is an empirical parameter related to the change in adsorption rate with surface coverage. For Weber–Morris, *k* represents the intraparticle diffusion rate constant, while *C* represents the intercept associated with boundary-layer resistance. In the Bangham model, *k*_B_ is the adsorption rate constant, and *Z* is the Bangham exponent, while *B*_*t*_ represents the Boyd time parameter. The *q*_e_ values reported in Table 4 represent the equilibrium adsorption capacities obtained as fitted parameters from nonlinear regression of the experimental kinetic data, rather than the experimentally measured CO_2_ uptake under the optimized operating conditions. Fig. 9 displays the nonlinear-fitting profiles alongside residual distribution plots, with the related kinetic parameters summarized in Table 4.

**Table 4:** Kinetic models comparison matrix

Model	*q*_e_ fit (mmol g^−1^)	Constants	*R* ^2^	AIC	ΔAIC
PSO	8.4973	*k* = 0.000537	0.9863	−726.74	146.22
Avrami	3.958	*k* = 0.01148, *n* = 1.31438	0.99401	−872.96	0
Elovich	—	*α* = 0.04022, *β* = 0.29609	0.98435	−702.92	170.04
PFO	5.218	*k* = 0.00721	0.98836	−756.03	116.93
Weber–Morris		*k* = 0.2656	0.9154	−428	444
Bangham model	3.96	*K*_B_ = 0.00287, *Z* = 1.30	0.984	−713	159
Boyds diffusion	3.30	*B* = 0.01	0.659	−264	608

**Fig. 9:**
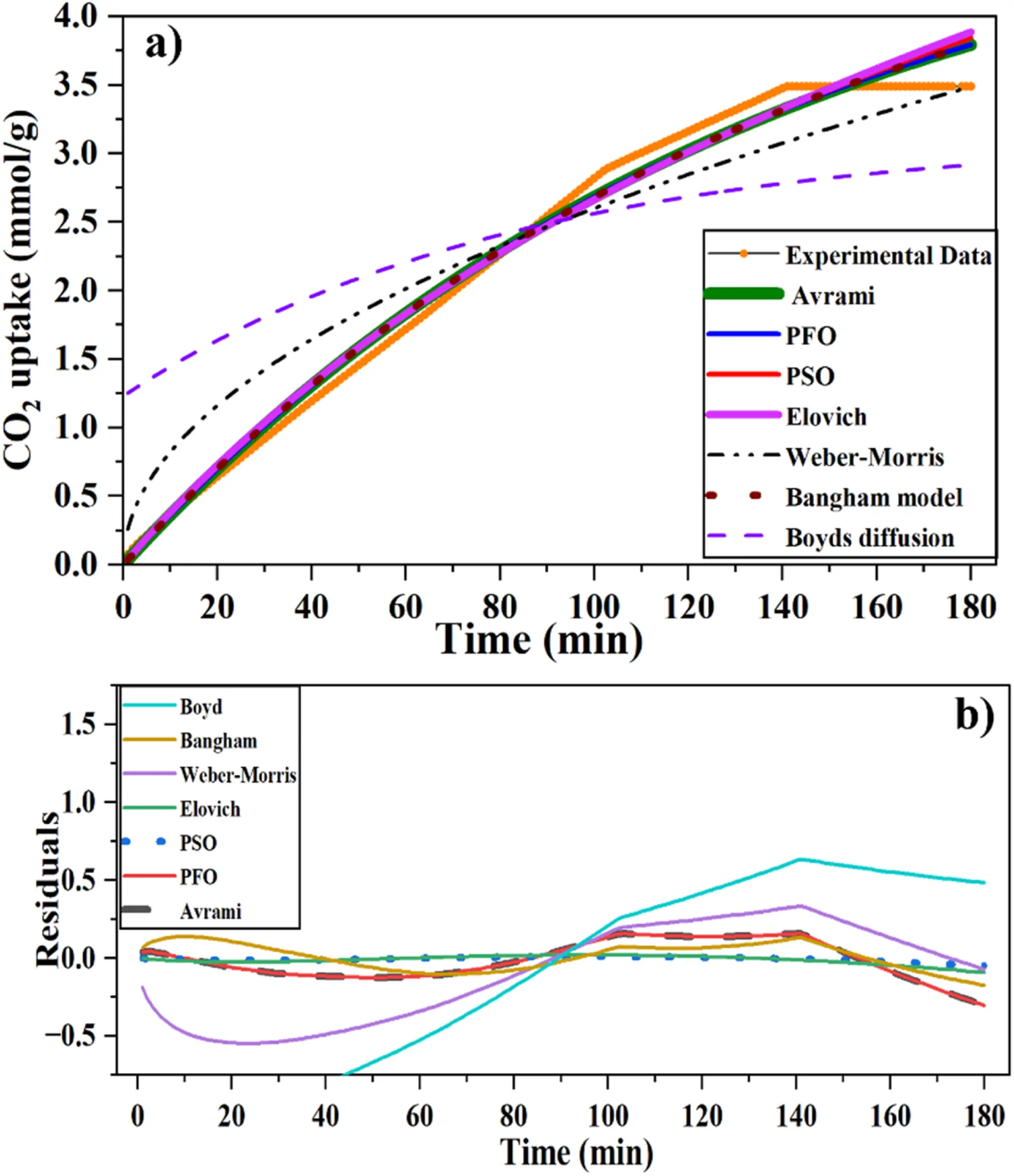
(a) Adsorption kinetic plots Zr-MOF-808-TETA (20%) fitted with various models and (b) residual plots (Δ*q*) indicating the goodness-of-fit.

The adsorption profile showed an initial uptake phase, followed by a slow approach to equilibrium. Such behaviour is commonly observed for microporous MOF-based adsorbents, where abundant vacant active sites are initially available for adsorption, resulting in fast adsorption kinetics during the early stage of adsorption. As adsorption progresses, the remaining active sites become progressively occupied, leading to a slower adsorption rate at longer contact times.^47^

Among the models evaluated, the Avrami model provided the best overall statistical description of the experimental uptake. It exhibited a high *R*^2^ of 0.994 and the lowest Akaike Information Criterion (AIC), indicating the best balance between goodness of fit and model complexity. The Avrami model statistically outperformed other kinetic models. This observation shows that considering only the *R*^2^ value is not sufficient when selecting a non-linear kinetic model. It also shows how important it is to use residual analysis and statistical values such as AIC together for a thorough evaluation of models.^51^

While the PFO, PSO, and Avrami models achieved high *R*^2^ values of 0.988, 0.986, and 0.994, respectively, their predicted *q*_e_ values differed notably. The measured CO_2_ uptake was 3.4 mmol g^−1^, whereas the PSO model estimated higher *q*_e_ values. This discrepancy arises because *q*_e_ from nonlinear regression is a model-dependent parameter indicating the asymptotic capacity of the fitted kinetic function, whereas the experimental value represents uptake within the observed timeframe. When the profile does not fully reach the asymptotic region, different kinetic models can fit the data well but yield significantly different *q*_e_ predictions. Similar inconsistencies between experimental and model-predicted CO_2_ uptake have been reported in previous CO_2_ adsorption studies.^53,54^ Consequently, the experimental uptake of 3.4 mmol g^−1^ was retained as the experimental adsorption capacity, while the kinetic models were used primarily to compare the temporal uptake behaviour and goodness of fit.^55,56^ The superior statistical fit of the Avrami model indicates that its empirical functional form describes the observed uptake profile more adequately than the other kinetic expressions considered. The Avrami exponent (*n* = 1.314) is an empirical fitting parameter describing the shape of the uptake profile. In humid conditions, water molecules may adsorb *via* bicarbonate pathways, which could contribute to the observed adsorption behaviour. Comparing the Avrami model with the PFO, PSO, Elovich, Weber–Morris, Bangham and Boyds diffusion models, we find that the residuals of the Avrami model are smaller and more uniformly distributed around zero, supporting its superior statistical fit. These findings align well with earlier research on amine-functionalized porous adsorbents based on MOF materials, in which the Avrami model provided the best fit. Additionally, 60P3Al, a PEI used to modify the adsorbent, also fits best with the Avrami model.^57^ Similarly, KA-30, a material derived from coal fly ash, demonstrated that the Avrami model best suited the kinetic analysis.^58^

### 4.4 Adsorption isotherm analysis

The adsorption behaviour of CO_2_ on TETA-functionalized Zr-MOF-808 was analysed using various isotherm models to understand the equilibrium characteristics of the adsorbent. The adsorption capacity was evaluated using the normalised CO_2_ partial pressure (*p*/*p*_0_) at constant temperature, with the fitted isotherms shown in Fig. 10. Adsorption was investigated by varying the CO_2_ partial pressure (0.1–0.8 bar) under optimal conditions. The Langmuir isotherm model represents monolayer adsorption on uniform sites with identical adsorption energy, whereas the Freundlich model describes adsorption on heterogeneous surfaces, which may include multilayer adsorption.^12^ Sips integrates aspects of both the Langmuir and the Freundlich models and provides an empirical representation of equilibrium adsorption over a range of adsorbate pressures.^59^ The Temkin isotherm describes adsorption on weakly interacting compounds and shows a linear decrease in the heat of adsorption.^60^ The equations for the Langmuir, Freundlich, Sips, and Temkin isotherms are provided below as eqn (9)–(12).^61^9
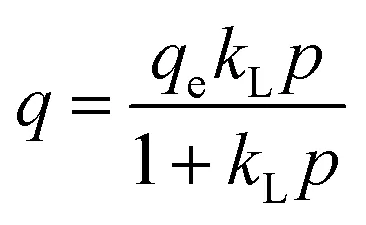
10
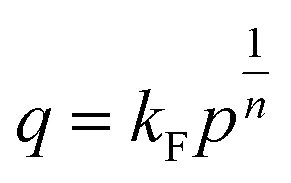
11
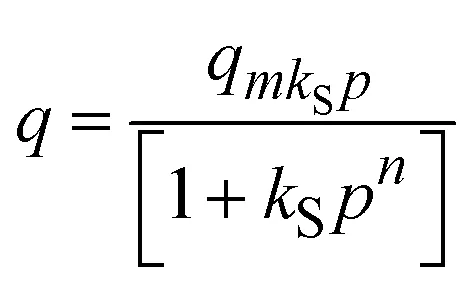

12 *q* = *B*(ln *K*_T_*P*_e_)

Here, *q*_*t*_ represents the adsorption capacity at time *t*, and *q*_e_ is the adsorption capacity at equilibrium. *k*_L_, *k*_S_ and *k*_F_ are Langmuir, Sips, and Freundlich adsorption constants. *B* is a temperature-dependent adsorption constant.

**Fig. 10:**
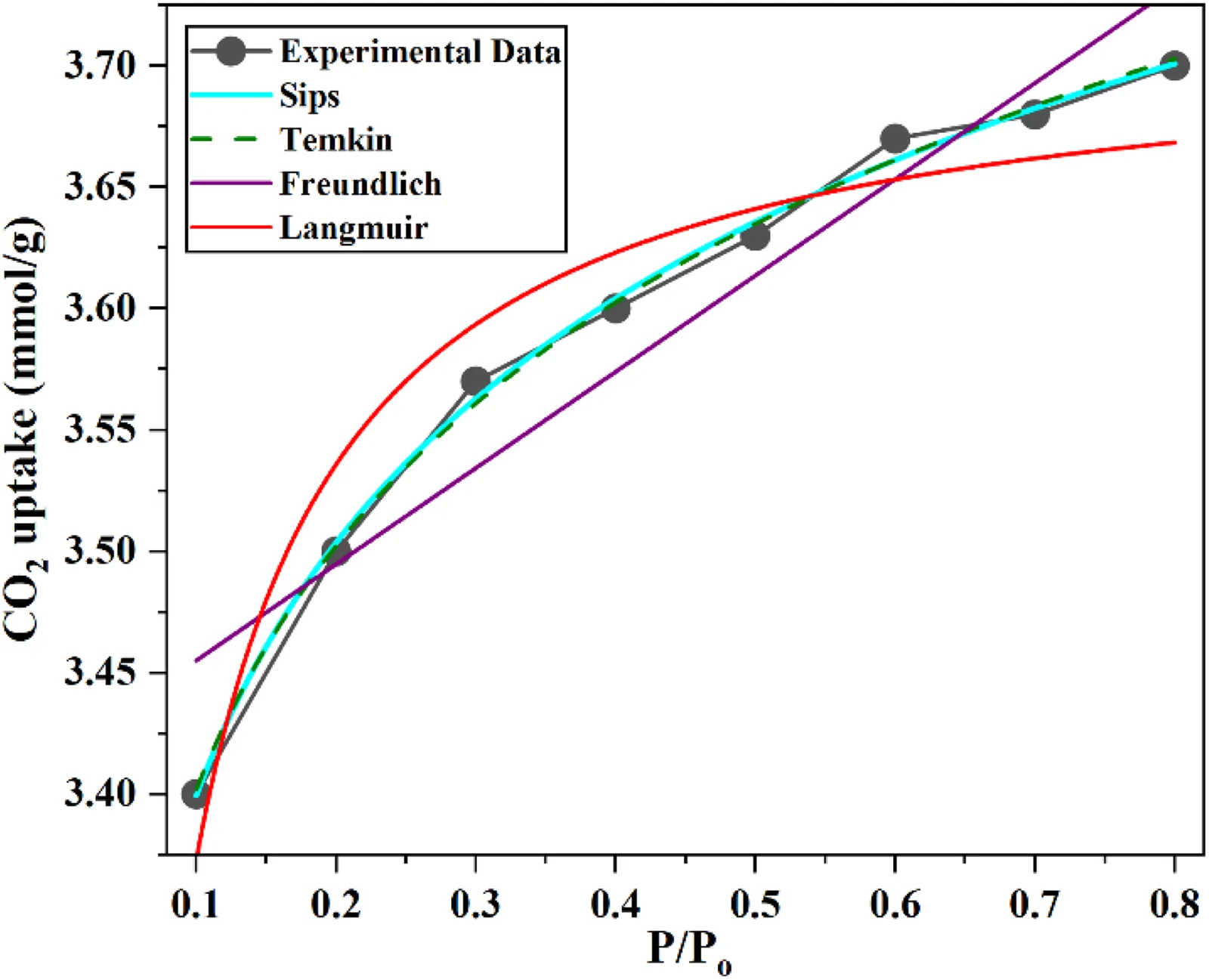
CO_2_ adsorption isotherm of Zr-MOF-808-TETA (20%).

The Sips model showed the best fit with *R*^2^ = 0.99744, followed closely by the Temkin model with *R*^2^ = 0.99664. The isotherm parameters are provided in Table 5. The Sips model offered the most accurate statistical description of the equilibrium data among the evaluated models, indicating it provided the most suitable empirical representation within the investigated pressure range. Although the Sips model can accommodate deviations from ideal homogeneous adsorption behaviour, the improved statistical fit alone cannot establish surface heterogeneity or a specific adsorption mechanism. Therefore, the Sips parameters are interpreted primarily as empirical descriptors of the equilibrium adsorption behaviour. A comparable study on amine-modified Mg–Al metal oxide also found that the Sips model best fits the equilibrium data.^62^

**Table 5:** Different parameters of isotherm models

Model	*q*_e_ (mmol g^−1^)	Constants	*R* ^2^
Temkin		*A* = 1.60, *B* = 0.144	0.996
Sips	5.09	*k*_S_ = 2.74, *n* = 0.135	0.997
Freundlich		*k*_F_ = 3.41, *n* = 2.52	0.906
Langmuir	3.71	*k*_L_ = 98.78	0.933

### 4.5 Mechanism of adsorption

The adsorption of CO_2_ onto TETA-functionalized Zr-MOF-808 was proposed to occur *via* a synergistic interaction between the porous zirconium-based framework and the nitrogen-rich amine functionalities introduced by TETA impregnation. Understanding adsorption mechanisms links an adsorbent's structure to its separation performance, which depends on both material properties and adsorbent–adsorbate interactions.^63,64^ XRD analysis showed that the Zr-MOF-808 framework retained its crystallinity after TETA impregnation, indicating that the addition of amine molecules does not compromise its structural integrity. Correspondingly, SEM analysis showed that the overall morphology of Zr-MOF-808 remained relatively unchanged after TETA functionalization; however, some agglomeration and increased roughness were observed as amine loading increased, suggesting deposition of TETA on the external surface, while BET analysis suggests partial occupation of the pore structure. The adsorption behaviour of a porous material is closely associated with its pore structure, surface characteristics, accessible functional groups, and molecular transport pathways. Modification of material surfaces can alter the accessibility of active sites and the interaction environment of adsorbed molecules.^65,66^

FTIR analysis identified distinct functional groups corresponding to TETA, indicating its successful incorporation. TETA reveals N–H stretching vibrations and enhances the lower-region bands associated with N–H and C–N bending vibrations.^39^ Incorporating appropriate functional groups into a porous substrate is an effective strategy for modifying chemical environments and introducing additional interaction sites. Such functionalization can alter the transport pathways and the material's affinity for the target species.^67^

These nitrogen groups are considered potential adsorption sites for CO_2_ and may facilitate acid–base interactions with the acidic CO_2_ molecules. Under dry conditions, CO_2_ adsorption may proceed through carbamate formation according to the following reaction.^40,68^

13 2RNH_2_ + CO_2_ ↔ RNHCOO^−^ + RNH_3_^+^ (carbamate formation)

Under humid conditions, water molecules may facilitate bicarbonate formation, which has been proposed to improve the efficiency of amine utilisation and lead to better CO_2_ adsorption performance. Though there is no direct spectroscopic evidence of bicarbonate species formation in this study, this pathway is suggested rather than definitively confirmed.

14 RNH_2_+ CO_2_ + H_2_O ↔ RNH_3_^+^ + HCO_3_^−^ (bicarbonate formation)

XPS further supports the presence of TETA through N 1s signals 399.5–399.8 eV in the TETA-modified samples.^69^ In addition, the persistence of the Zr 3d and O 1s peaks confirms that zirconium oxo clusters remained chemically stable following functionalization. The BET analysis shows that increasing TETA loading decreases pore volume and surface area, as amine molecules partially fill the pores. The mass loss between 250 °C and 300 °C is attributed to thermal degradation of TETA. Despite functionalization, the material retained relatively good thermal stability, indicating that the zirconium-based framework remained sufficiently robust during adsorption and regeneration.

Overall, the mechanism of CO_2_ adsorption onto TETA-functionalized Zr-MOF-808 is proposed to involve initial pore diffusion and subsequent adsorption within the porous MOF framework, followed by amine-assisted adsorption *via* a possible carbamate under dry conditions and bicarbonate formation under humid environments. The following mechanism is picturized in Fig. 11.

**Fig. 11:**
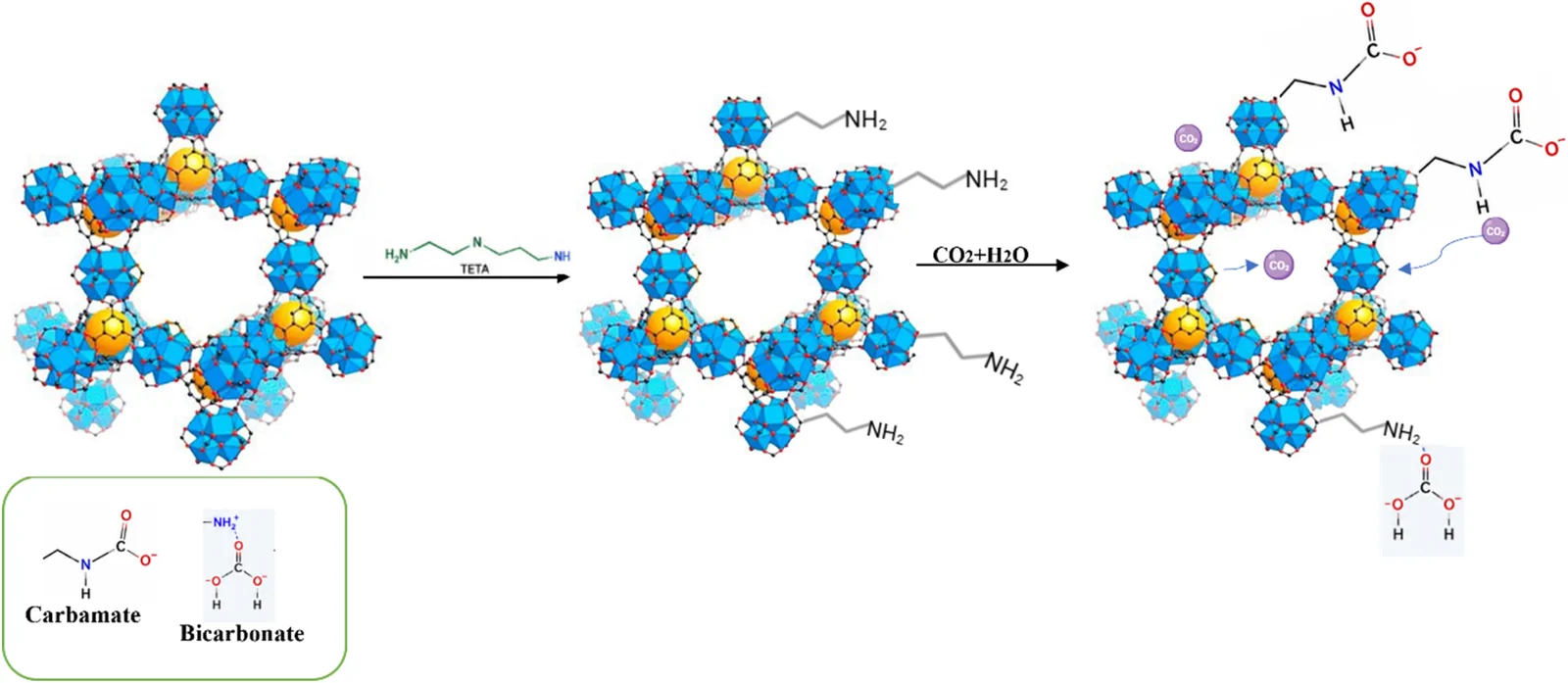
Illustration of the adsorption mechanism of CO_2_ on TETA-functionalized Zr-MOF-808, showing TETA functionalization with possible carbamate/bicarbonate formation.

## 5 Cost analysis

The total synthesis cost of Zr-MOF-808-TETA (20%) is determined using the following equation, which accounts for both raw material and energy expenses per kilogram product.^70^15
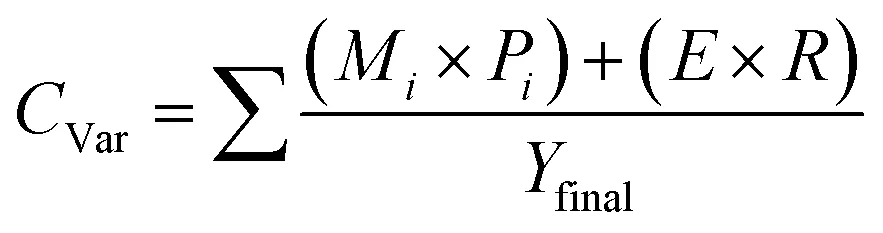
Here, *M*_*i*_ (kg) is the amount of precursor; *P*_*i*_ ($ per kg) is the amount; *E* (k Wh) is the amount of energy consumed; *Y* (kg) is the amount of adsorbent after synthesis; *R* is the cost of electricity.

The lab-scale synthesis cost of Zr-MOF-808-TETA (20%) is ₹114 285 per kg, converted to $1182 per kg. However, it still costs more than conventional adsorbents (activated carbon, $0.50–$2.00 per kg; zeolite 13X, $1.00–$3.00 per kg). The higher cost mainly results from research-grade chemicals, small-batch synthesis, and solvent-heavy processing. Costs could drop significantly with bulk raw materials, solvent recycling, process intensification, and large-scale continuous manufacturing. The detailed cost analysis is mentioned in the SI data Table S5.

### 5.1 End-of-life repurposing

Over multiple cycles, CO_2_ uptake by the spent adsorbent diminishes due to pore blockage and structural breakdown.^71^ Instead of disposing of the MOF through conventional land-filling, it can be reused for other applications, as landfilling may lead to secondary pollution and ecotoxicological effects if not properly managed. One way to reuse spent sorbent is to create fired clay bricks; adding spent sorbent to clay increases compressive strength from approximately 10.8 MPa to 15.8 MPa when it accounts for about 25% of the total mass.^72^ Moreover, even though the spent Zr-MOF adsorbents are no longer capable of capturing CO_2_, they can still be used as catalysts, since the properties of both the porous structure and the metal nodes of the framework can be leveraged for further chemical conversion of captured CO_2_ into other final materials, such as methanol.^73^ Finally, the spent MOFs can also be converted into carbon-based materials through thermal treatment, thereby enabling entirely different uses of the same raw material. A Zn-based MOF has been used to produce a hierarchically porous, nitrogen-doped carbon nanofiber capable of degrading organic pollutants in water treatment applications,^74^ while separately, MOF-derived carbon composites have been reported as effective electrode materials for supercapacitors, leveraging the high porosity and tunable pore architecture retained from the parent MOF structure.^75^ All these routes suggest that used amine-modified Zr-MOF adsorbents can be converted from waste into construction-quality materials, catalysts, water-cleaning absorbents, or electrodes in energy storage systems, thereby immobilising or repurposing residual amine and metal species while supporting circular-economy handling of end-of-life CO_2_ capture materials.^72^

## 6 Cyclic stability

To assess the regeneration capability and structural integrity of the synthesized adsorbent, its cyclic stability was evaluated over 10 cycles under humid conditions. Desorption was performed at 120 °C for 1 hour to avoid amine leaching and framework degradation at higher temperatures.^76^ As shown in Fig. 10, the adsorbent's CO_2_ uptake remained fairly stable over 10 cycles, with only a gradual decline in adsorption capacity. This initial CO_2_ uptake capacity of approximately 3.4 mmol g^−1^ gradually decreased to nearly 3.0 mmol g^−1^ after the tenth cycle, corresponding to a retention of approximately 88%. The cyclic retention capacity of the adsorbent is shown in Fig. 12.

**Fig. 12:**
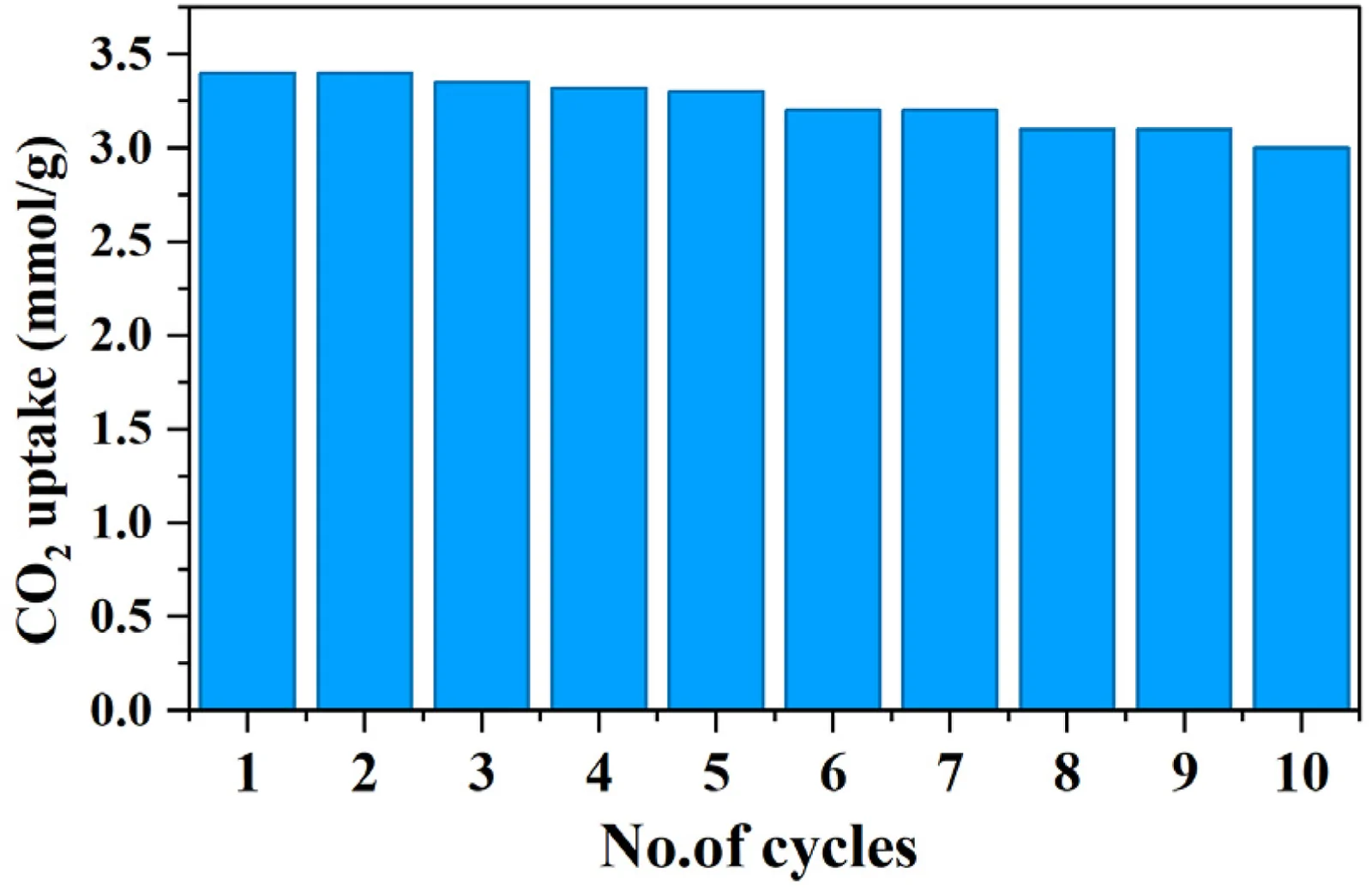
Cyclic stability of Zr-MOF-808-TETA (20%).

The drop in adsorption efficiency over repeated cycles may be attributed to partial pore clogging by residual adsorbed CO_2_/H_2_O molecules and minor diffusion limitations.^77^ The XRD, FTIR, and SEM images of both the fresh and spent adsorbents are shown in Fig. 13. From both the XRD and SEM images, it is evident that the material shows no significant structural degradation. The XRD pattern of the spent adsorbent showed peak broadening and reduced intensities, but retained all the prominent peaks of the fresh adsorbent. However, no additional peaks were observed after 10 cycles, indicating that no structural transformation had occurred. In SEM, an agglomeration of particles is evident, blocking pores compared to the fresh sample. In FTIR, both samples showed prominent M–O peaks at 500–700 cm^−1^, C–N stretch at 1300–1400 cm^−1^, N–H bending at 1550–1620 cm^−1^, and broad O–H/N–H stretch at 3300–3600 cm^−1^. A new weak shoulder peak is observed near 1710 cm^−1^, which may be attributed to carbamic acid or hydrogen-bonded carbamate after regeneration. Overall, these characterization techniques show that the core crystalline structure remained intact after 10 cycles. However, peak broadening, decreased XRD intensity, and particle agglomeration indicate minor structural changes. Results suggest structure and CO_2_ uptake are maintained over 10 cycles, but longer-term testing is needed to confirm long-term stability.

**Fig. 13:**
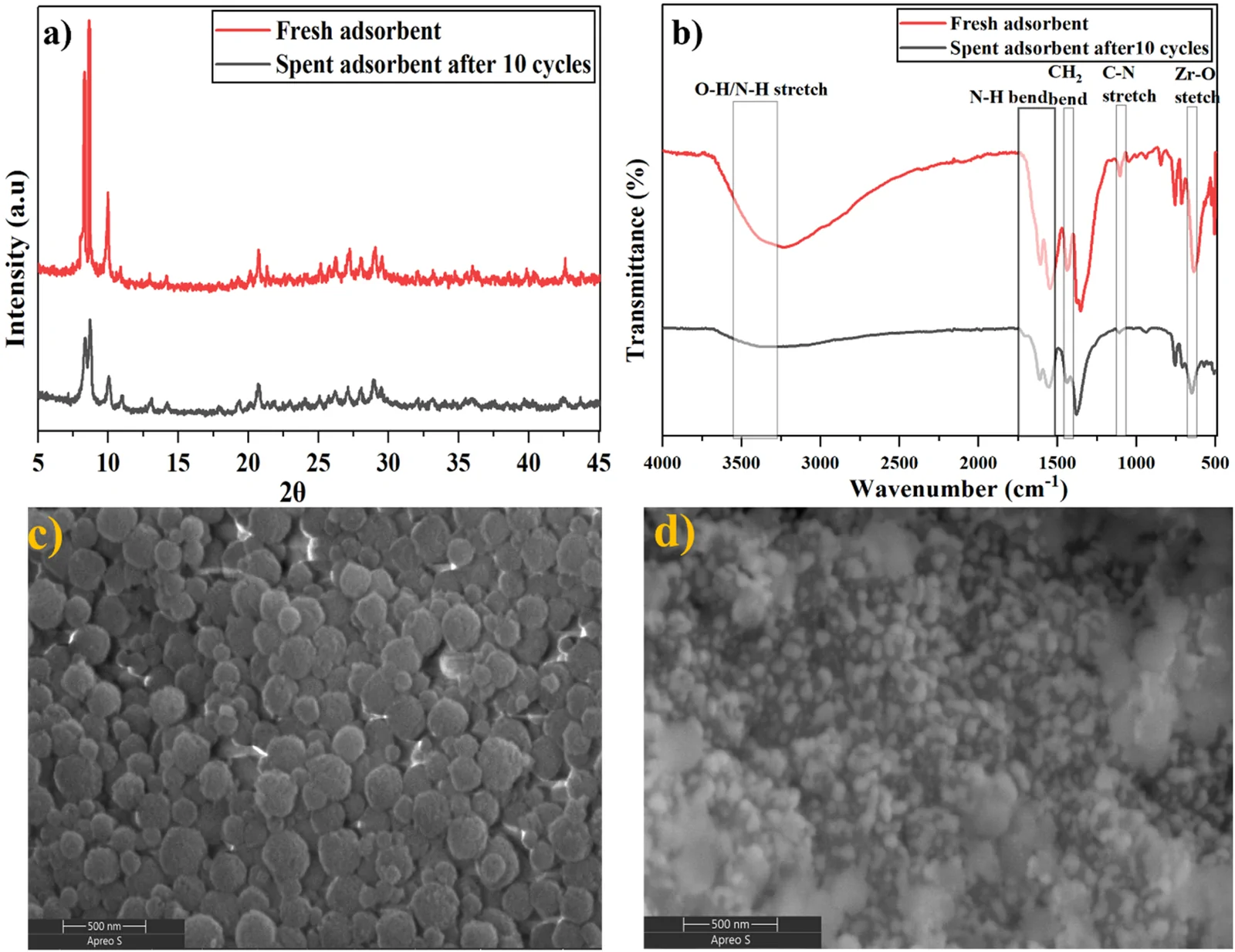
(a) XRD of both fresh and spent adsorbent, (b) FTIR of both fresh and spent adsorbent, (c) and (d) SEM images of both fresh and spent adsorbent.

Table 6 compares this work with the reported adsorbents.

**Table 6:** Cyclic stability comparison

S. no.	Name of the adsorbent	CO_2_ adsorption capacity (mmol g^−1^)		Surface area (m^2^ g^−1^)	No. of cycles	% of drop in CO_2_ adsorption capacity	Ref.
		Dry	Humid				
1	30% TEPA/SiO_2_	—	1.89	69	10	4.23	78
2	30% TEPA+ 30% DEA/SiO_2_	—	4.14	15	5	4.14	78
3	MCNTs/TEPA	3.32	5.0	2.2	10	20	79
4	MOF-199	3.3	2.5	1176	10	27	80
5	Zeolite-13X	3.9	3.3	688	10	24	80
6	MOF-808	2.17	0.69	1181.14	5	No loss	81
7	F-MOF-808	1.97	1.72	10.32.75	5	No loss	81
8	KIT-6-TEPA	2.84	3.2	—	10	1.5	82
9	Zr-MOF-808-TETA (20%)	—	3.4	142	10	12	This work

## 7 Conclusion

This study shows that modifying materials with amines successfully enhances CO_2_ adsorption under humid environments. Here, 20 wt% functionalized Zr-MOF-808 exhibited good capture capacity of 3.4 mmol g^−1^ under optimal conditions, *viz.*, 55 °C, 20 wt% TETA, 3 g, and 180 minutes, as determined using the RSM-CCD method. The Avrami model best described the kinetic data, while the Sips model accurately described the equilibrium data. The adsorbent retained 88% of its original uptake capacity over 10 consecutive runs, demonstrating stable performance over the tested cycling period. These findings suggest that TETA-functionalized Zr-MOF-808 holds potential for humid carbon capture. Future work should include developing composites, applying hydrophobic surface modifications, and performing extended cyclic tests to assess performance in humid flue gas conditions.

## Author contributions

Neha Mohiuddin: methodology, writing – original draft. Akshit Kumar: resources and funding. Rajesh Yadav: resources and funding. Satyapaul A. Singh: supervise, review, and editing. I. Sreedhar: supervise, conceptualize, and write – review & editing.

## Conflicts of interest

There are no conflicts to declare.

## Abbreviations

A with respect to RSM: Temperature (°C)
AIC: Akaike information criterion
Amine loading: Amine loading (wt%)
*B*
: Amount of adsorbent (g)
*C*
: Carbonation time (min)
CC: Carbon capture
CCD: Central composite design
CO_2_ adsorption: CO_2_ adsorption capacity (mmol g^−1^)
CCUS: Carbon capture utilization and storage
*D*
: % amine of loading (wt%)
DMF: Dimethyl formamide
*E*
: Amount of energy consumed (kWh)
*q*
: Flow rate (ml min^−1^)
*k*
: Respective kinetic and isotherm constant
*M*_*i*_
: Amount of precursor (kg)
MOF: Metal–organic framework
*Q*_e_
: CO_2_ uptake at equilibrium (mmol g^−1^)
*Q*_*t*_
: CO_2_ uptake at time (*t*) (mmol g^−1^)
RH: Relative humidity (%)
RSM: Response surface methodology (RSM)
*T*
: Temperature (°C)
TETA: Triethylenetetramine (TETA)
*t*
: Time (min)
*Y*
: Amount of adsorbent obtained after synthesis (kg)

## Supplementary Material

RA-016-D6RA07412J-s001

## Data Availability

The data supporting this article are included within the article and in the supplementary information (SI). Supplementary information: synthesis protocols, physico-chemical characterization results, experimental design through RSM with model fitting and cost analysis. See DOI: https://doi.org/10.1039/d6ra07412j.
