# Supplementary material for: Efficient and stable carbon capture from industrial flue gas under humid conditions using amine-functionalized Zr-MOF-808 through statistical process design
Source: RSC Adv. 2026 Oct 9;16(47):61851–65. doi: 10.1039/d6ra07412j (PMC13650933; doi:10.1039/d6ra07412j)
Supplement: Supplementary file 1 [file RA-016-D6RA07412J-s001.pdf]

This journal is © The Royal Society of Chemistry

Supplementary Data

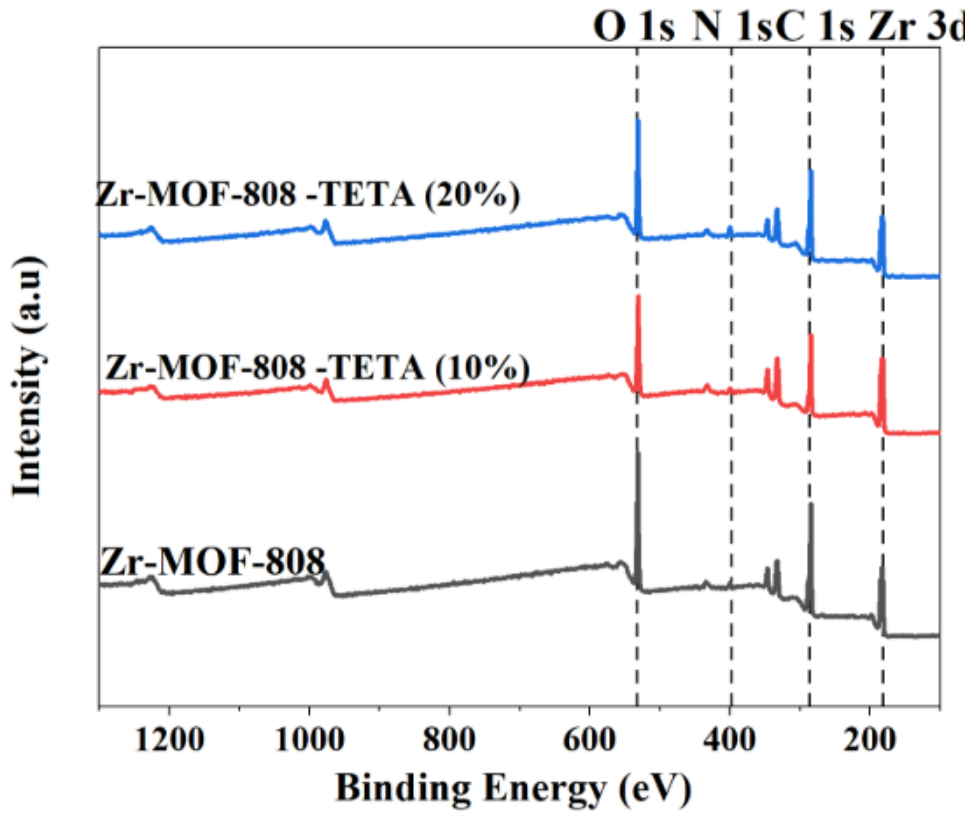

Figure S.1. XPS survey spectra of pristine and amine-modified Zr-MOF-808.

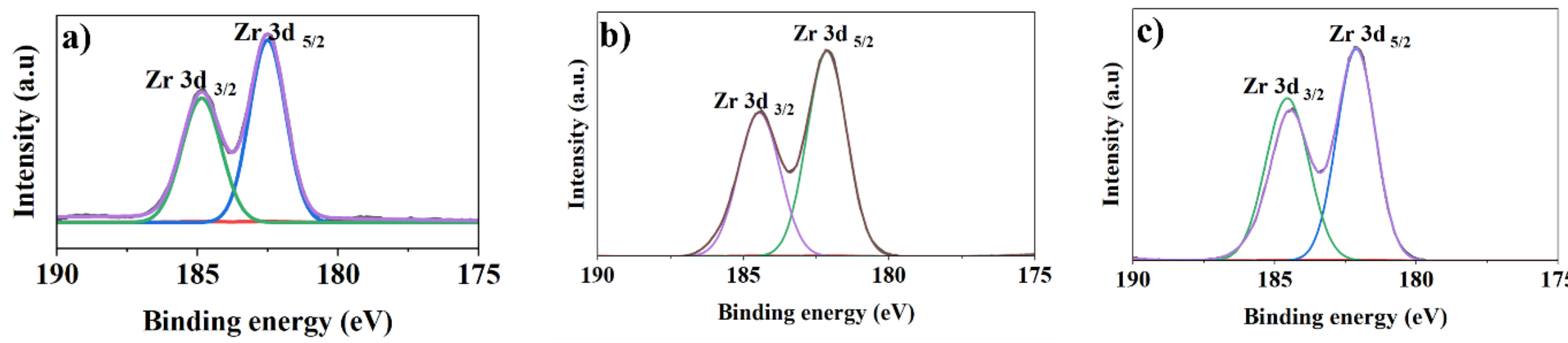

Figure S.2. Zr 3d deconvoluted spectra of a) Zr-MOF-808, b) and c) Zr-MOF-808-TETA (10 % and 20%)

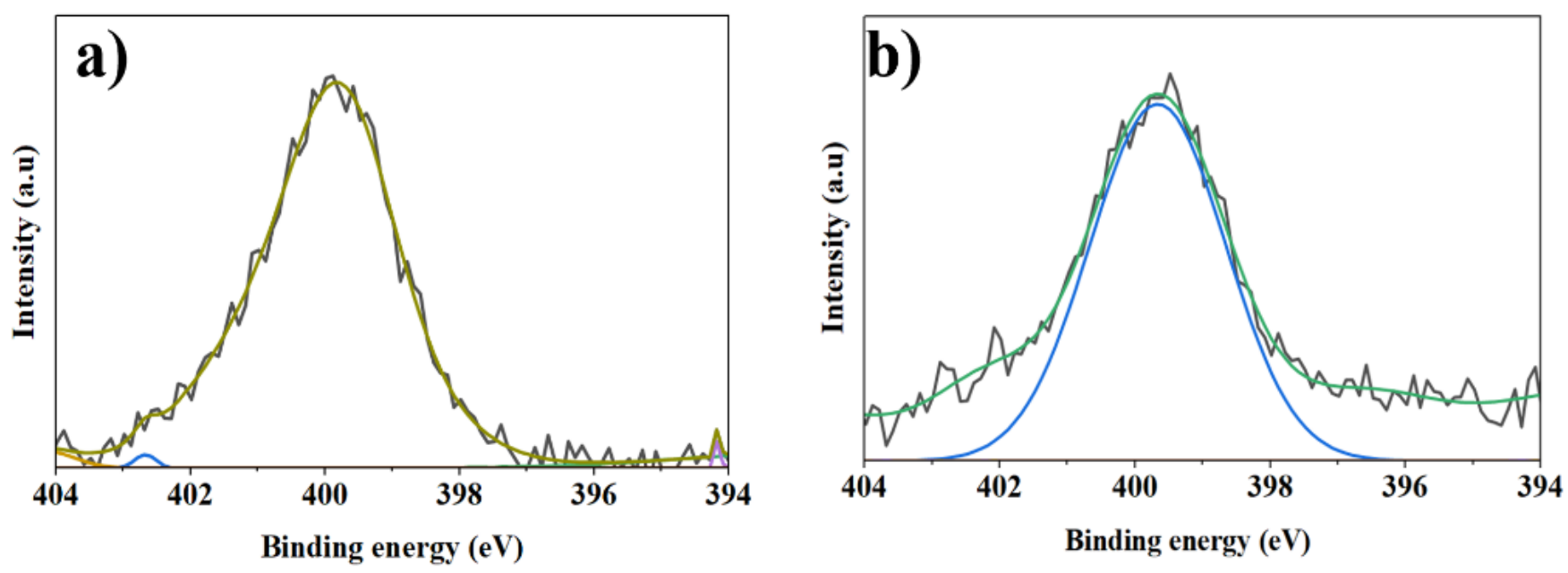

Figure S.3. N 1s deconvoluted spectra of a) and b) Zr-MOF-808-TETA (10 % and 20%)

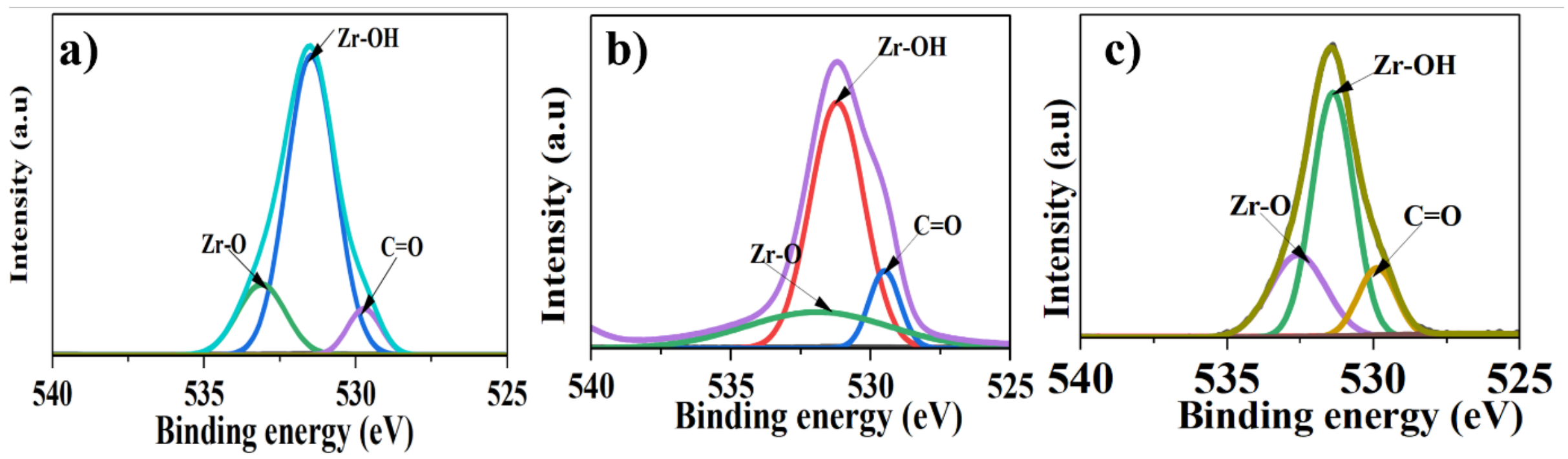

Figure S.4. O 1s deconvoluted spectra of a) Zr-MOF-808, b) and c) Zr-MOF-808-TETA (10 % and 20%)

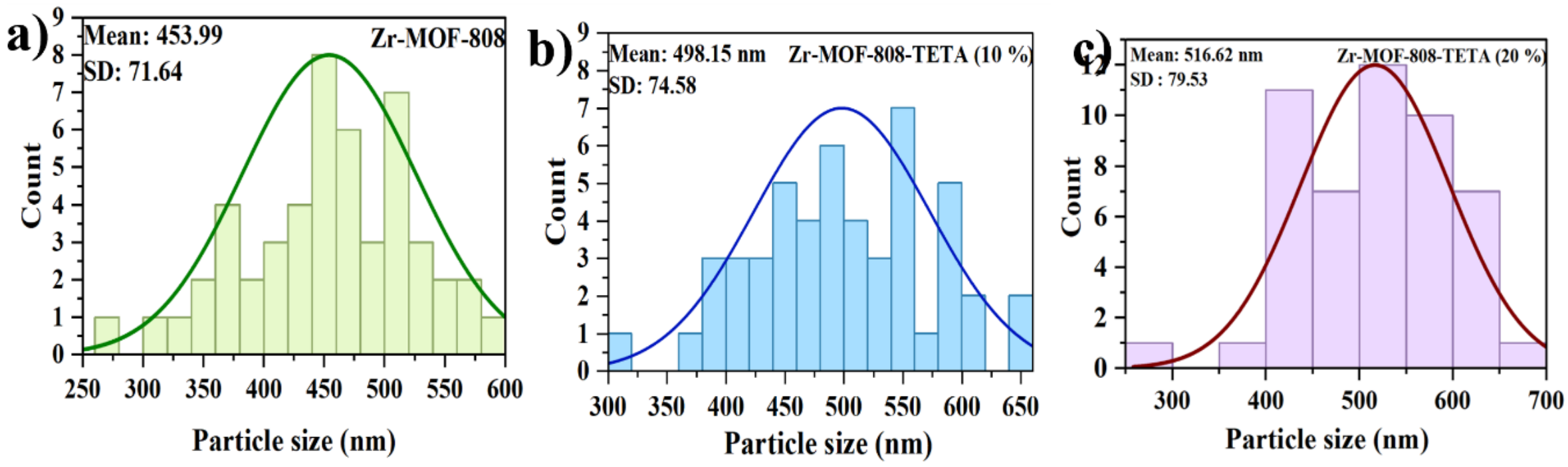

Figure S.5. Particle size distribution of (a) pristine Zr-MOF-808, (b) Zr-MOF-808-TETA (10%), and (c) Zr-MOF-808-TETA (20%)

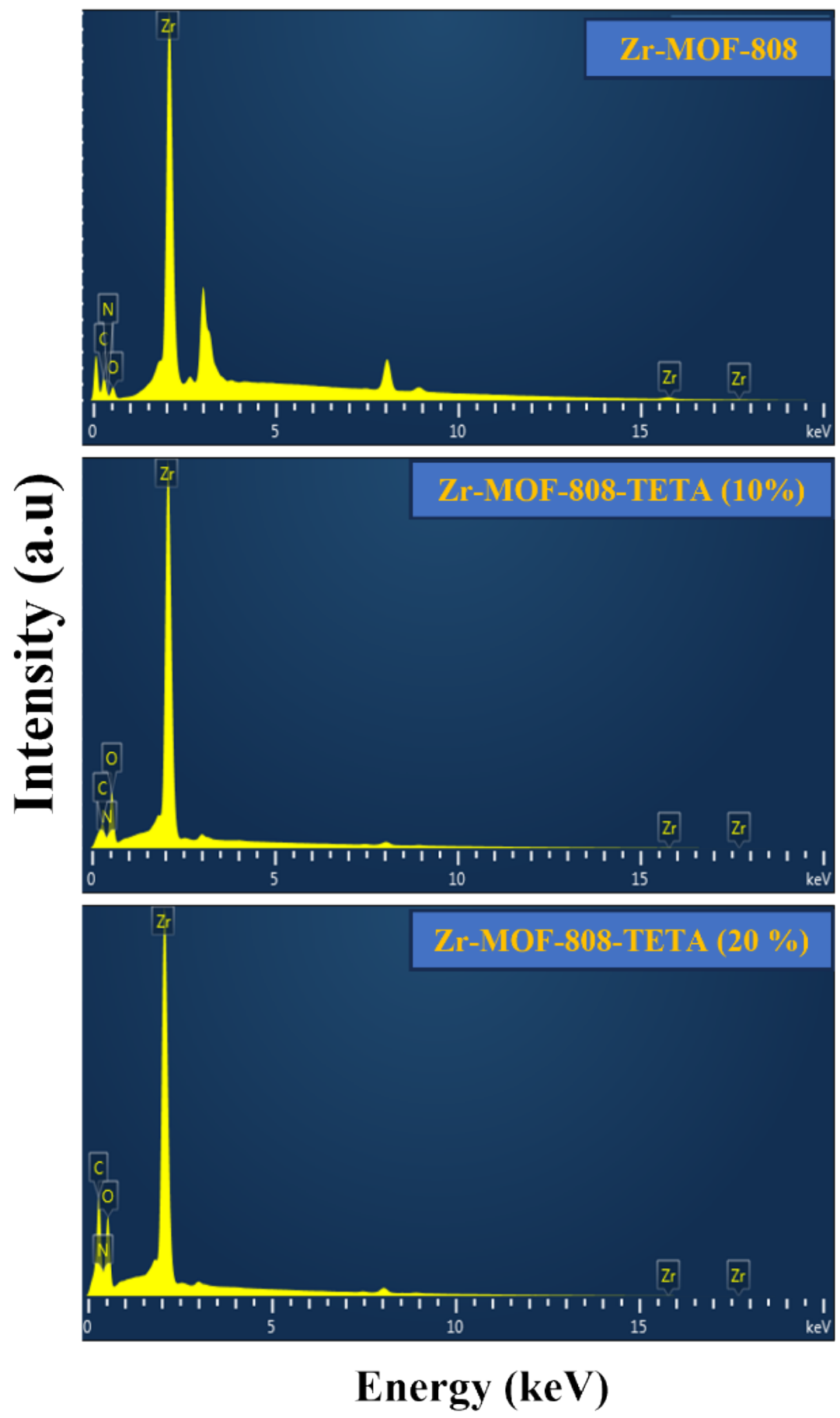

FigureS.6. EDS spectra showing different elements present in pristine and amine-modified Zr-MOF-808.

Table.S.1. Elemental composition of ZIF-8 and Cu-Zn-ZIF-8 as measured by using EDS.

EDS analysis	Weight (%)			
Elements	Zr	N	O	C
Zr-MOF-808	59.29	0.03	8.10	32.59
Zr-MOF-808-TETA (10%)	51.46	2.71	24.62	21.20
Zr-MOF-808-TETA (20%)	30.93	5.90	22.38	40.78

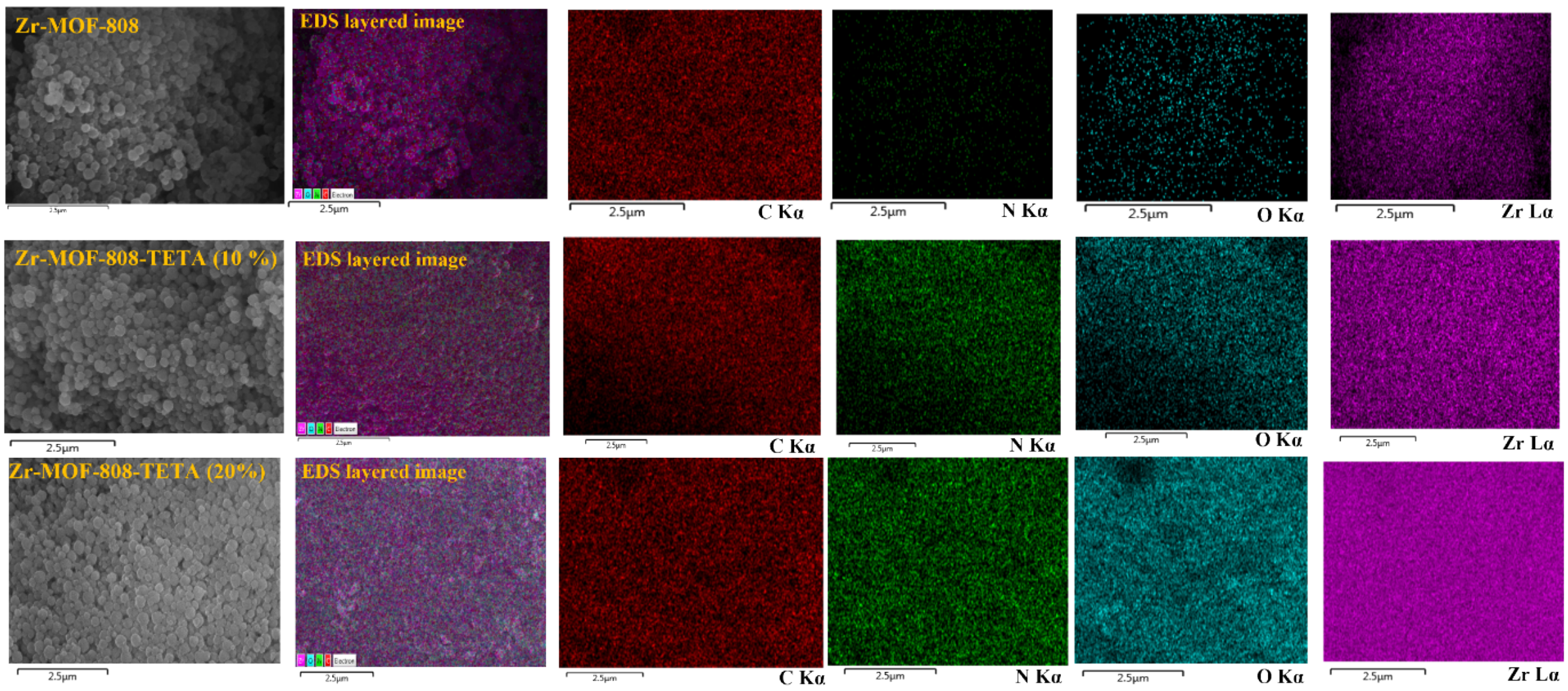

Figure S.7. EDS layered overlays, and corresponding elemental mapping profiles of (a) pristine Zr-MOF-808, (b) Zr-MOF-808-TETA (10%), and (c) Zr-MOF-808-TETA (20%) tracking Carbon (C Kα, red), Nitrogen (N Kα, green), Oxygen (O Kα, cyan), and Zirconium (Zr Lα, purple). Scale bars represent 2.5 μm.

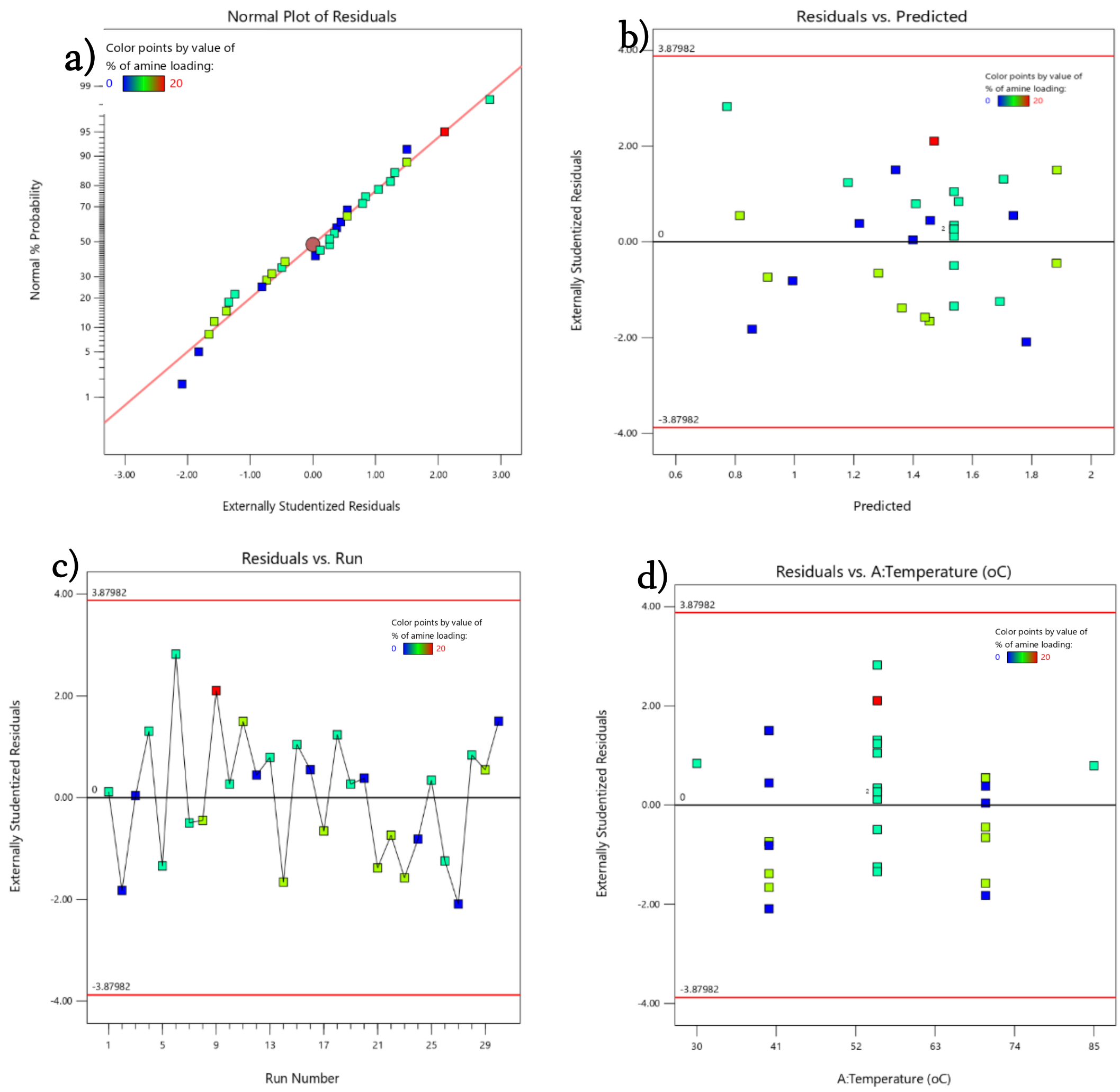

Figure S.8. Residual plots for the quadratic model assessing adsorption capacity: (a) Normal probability plot, (b) residuals v/s predicted values, (c) residuals plotted against run order, and (d) residuals compared to Factor A (Temperature).

Table S.2. Experimental design of RSM:

Run	A: Temperature (° C)	B: Amount of adsorbent (g)	C: Time (min)	D: Amine loading (wt %)	CO_2 adsorption capacity (mmol/g)
1	55	5	120	10	2.38
2	70	7	60	0	0.66
3	70	7	180	0	1.96
4	55	5	240	10	3
5	55	5	120	10	2.28
6	55	10	120	10	0.64
7	55	5	120	10	2.3
8	70	3	180	20	3.3

9	55	5	120	30	2.28
10	55	5	120	10	2.4
11	40	3	180	20	3.7
12	40	7	180	0	2.16
13	85	5	120	10	2.04
14	40	7	180	20	1.99
15	55	5	120	10	2.5
16	70	3	180	0	3.07
17	70	3	60	20	1.6
18	55	5	60	10	1.51
19	55	5	120	10	2.4
20	70	3	60	0	1.51
21	40	3	60	20	1.76
22	40	7	60	20	0.79
23	70	7	180	20	1.95
24	40	7	60	0	0.94
25	55	5	120	10	2.41
26	55	3	120	10	2.7
27	40	3	180	0	3
28	30	5	120	10	2.5
29	70	7	60	20	0.69
30	40	3	60	0	1.9

Table S.3. ANOVA Table:

**Source**	**Sum of Squares**	**df**	**Mean Square**	**F-value**	**p-value**	
**Model**	2.61	14	0.1862	94.32	< 0.0001	significant
A-Temperature	0.0024	1	0.0024	1.23	0.2850	
B-Amount of adsorbent	0.7101	1	0.7101	359.63	< 0.0001	
C-Time	0.2476	1	0.2476	125.39	< 0.0001	
D-Amine loading %	0.0000	1	0.0000	0.0221	0.8838	
AB	0.0002	1	0.0002	0.1060	0.7493	
AC	0.0061	1	0.0061	3.09	0.0990	
AD	0.0018	1	0.0018	0.9368	0.3485	
BC	0.0005	1	0.0005	0.2739	0.6084	

BD	0.0111	1	0.0111	5.64	0.0313	
CD	0.0067	1	0.0067	3.41	0.0846	
A^2	0.0049	1	0.0049	2.47	0.1366	
B^2	0.0561	1	0.0561	28.38	< 0.0001	
C^2	0.1322	1	0.1322	66.94	< 0.0001	
D^2	0.0095	1	0.0095	4.80	0.0446	
**Residual**	0.0296	15	0.0020			
Lack of Fit	0.0238	9	0.0026	2.74	0.1160	not significant
Pure Error	0.0058	6	0.0010			
**Cor Total**	2.64	29				

Table S.4. Fit statistics

**Std. Dev.**	**R^2**	**Adjusted R^2**	**Predicted R^2**	**C.V%**
0.0444	0.9888	0.9783	0.8933	3.15

Table S.5. Coefficients in Terms of Coded Factors

**Factor**	**Coefficient Estimate**	**df**	**Standard Error**	**95% CI Low**	**95% CI High**	**VIF**
Intercept	1.48	1	0.0169	1.45	1.52	
A-Temperature	-0.0232	1	0.0209	-0.0678	0.0214	2.84
B-Amount	-0.4527	1	0.0239	-0.5035	-0.4018	2.18
C-Time	0.2590	1	0.0231	0.2097	0.3083	2.52
D-% of amine loading	0.0023	1	0.0152	-0.0301	0.0346	2.32
AB	-0.0084	1	0.0259	-0.0637	0.0468	1.96
AC	0.0391	1	0.0222	-0.0083	0.0864	1.50
AD	0.0143	1	0.0148	-0.0172	0.0459	1.14
BC	0.0153	1	0.0292	-0.0469	0.0774	2.10
BD	-0.0462	1	0.0194	-0.0876	-0.0047	1.70

CD	0.0308	1	0.0167	-0.0047	0.0663	1.31
A^2	-0.0267	1	0.0170	-0.0629	0.0095	1.70
B^2	-0.1327	1	0.0249	-0.1858	-0.0796	1.67
C^2	-0.2055	1	0.0251	-0.2591	-0.1520	1.68
D^2	-0.0245	1	0.0112	-0.0483	-0.0007	1.18

2. Cost analysis:

2.1 Lab scale:

Energy and solvent usage were normalized to industrial-scale estimates rather than direct linear scaling of lab-scale values.

Lab-scale aqueous synthesis

- Trimesic acid = 0.34 g
- Total mass of salt = 1.04g
- Yield = 0.7 g (assuming 70% effective yield)
- Methanol wash = 100ml
- Assumed Industrial Energy Intensity = 30kWh

Cost Basis: Lab-grade materials are sourced from the SRL catalogue as of May 2026.

Table.S.5

Chemical	Amount	Cost Basis (₹/kg)	Effective Cost (₹)
Trimesic acid	0.3125 g	42000	13.23
Zirconium oxy chloride	0.727 g	3300	2.40
Formic acid	33.7 ml	1120/L	37.74
DMF	33.7 ml	600/L	20.22
TETA	0.16g	650	0.104
Methanol (washing, 95% recovery)	100 ml (79 g)	80	6.3
Total			₹ 79.994

Average yield: 70 % effective yield (0.7g)

Cost per kg adsorbent (lab scale): ₹80 / 0.0007 kg = ₹1,14,285/kg

This lab-scale cost is dominated by the linker and solvents. Significant cost reductions are expected through bulk procurement and process optimization at industrial scale.
